# National sex- and age-specific burden of blindness and vision impairment by cause in Mexico in 2019: a secondary analysis of the Global Burden of Disease Study 2019

**DOI:** 10.1016/j.lana.2023.100552

**Published:** 2023-07-11

**Authors:** Sofia E. Madueña-Angulo, Saul A. Beltran-Ontiveros, Emir Leal-Leon, Jose A. Contreras-Gutierrez, Erik Lizarraga-Verdugo, Perla Y. Gutierrez-Arzapalo, Silvia Lizarraga-Velarde, Efrain Romo-Garcia, Jesus Montero-Vela, Jose M. Moreno-Ortiz, Noemi Garcia-Magallanes, Hector M. Cuen-Diaz, Javier Magaña-Gomez, Diana Z. Velazquez, Pavel E. Hernandez-Carreño, Francisco Jimenez-Trejo, Mariana Reyes, Frida P. Muñiz, Daniel Diaz

**Affiliations:** a Especialidad en Oftalmología, Centro de Investigación y Docencia en Ciencias de la Salud, Universidad Autónoma de Sinaloa, Culiacán Rosales, 80030, Sinaloa, Mexico; b Centro de Investigación y Docencia en Ciencias de la Salud, Universidad Autónoma de Sinaloa, Culiacán Rosales, 80030, Sinaloa, Mexico; c Laboratorio de Genética y Biología Molecular, Facultad de Ciencias Químico Biológicas, Universidad Autónoma de Sinaloa, Culiacán, 80010, Mexico; d Instituto de Genética Humana “Dr. Enrique Corona Rivera”, Centro Universitario de Ciencias de la Salud, Universidad de Guadalajara, Guadalajara, 44340, Jalisco, Mexico; e Laboratorio de Biomedicina y Biología Molecular, Unidad Académica de Ingeniería en Biotecnología, Universidad Politécnica de Sinaloa, 82199, Mazatlán, Sinaloa, Mexico; f Laboratorio de Nutrición Molecular, Escuela de Nutrición y Gastronomía, Universidad Autónoma de Sinaloa, Culiacán, Sinaloa, Mexico; g Departamento de Biomedicina Molecular, Centro de Investigación y de Estudios Avanzados, Gustavo A. Madero, 07360, Ciudad de México, Mexico; h Departamento de Salud Pública, Facultad de Medicina Veterinaria y Zootecnia, Universidad Nacional Autónoma de México, 04510, Ciudad de México, Mexico; i Laboratorio de Morfología Celular y Tisular, Instituto Nacional de Pediatría, Ciudad de México, 04530, Mexico; j Doctorado en Ciencias Médicas, Odontológicas y de la Salud, Universidad Nacional Autónoma de México, Coyoacán, 04510, Ciudad de México, Mexico; k Doctorado en Ciencias Biomédicas, Universidad Nacional Autónoma de México, Coyoacán, 04510, Ciudad de México, Mexico; l Centro de Ciencias de la Complejidad (C3), Universidad Nacional Autónoma de México, Coyoacán, 04510, Ciudad de México, Mexico

**Keywords:** Avoidable blindness, Disease burden, Public health, Vision loss

## Abstract

**Background:**

Reliable national estimations for blindness and vision impairment are fundamental to assessing their burden and developing public health policies. However, no comprehensive analysis is available for Mexico. Therefore, in this observational study we describe the national burden of blindness and vision loss by cause and severity during 2019.

**Methods:**

Using public data from the Global Burden of Disease (GBD) study 2019, we present national prevalence and years lived with disability (YLDs) counts and crude and age-standardized rates (per 100,000 people) of total, severity- and cause-specific blindness and vision impairment with 95% uncertainty intervals (UIs) by sex and age group.

**Findings:**

In Mexico, the burden of blindness and vision impairment was estimated at 11.01 million (95% UI, 9.25–13.11) prevalent cases and 384.96 thousand (259.57–544.24) YLDs during 2019. Uncorrected presbyopia caused the highest burden (6.06 million cases, 4.36–8.08), whereas severe vision loss and blindness affected 619.40 thousand (539.40–717.73) and 513.84 thousand (450.59–570.98) people, respectively. Near vision loss and refraction disorders caused 78.7% of the cases, whereas neonatal disorders and age-related macular degeneration were among the least frequent. Refraction disorders were the main cause of moderate and severe vision loss (61.44 and 35.43%), and cataracts were the second most frequent cause of blindness (26.73%). Females suffered an overall higher burden of blindness and vision impairment (54.99% and 52.85% of the total cases and YLDs), and people >50 years of age suffered the highest burden, with people between 70 and 74 years being the most affected.

**Interpretation:**

Vision loss represents a public health problem in Mexico, with women and older people being the most affected. Although the causes of vision loss contribute differentially to the severity of visual impairment, most of the impairment is avoidable. Consequently, a concerted effort at different levels is needed to alleviate this burden.

**Funding:**

This study received no funding.

**Research in context**

**Evidence before this study**

In September 2022, we performed electronic database searches in PubMed to identify published studies reporting the epidemiology of vision impairment by severity and causes at the national level for Mexico. We included the following search terms: (blindness OR vision loss OR vision impairment OR visually impaired OR avoidable blindness) AND (refraction disorders OR cataract OR glaucoma OR diabetic retinopathy OR presbyopia OR near vision loss OR age-related macular degeneration) AND (Mexico OR Mexican population). Although we found 18 relevant studies that reported the prevalence of blindness and vision loss for some causes, these studies were performed at a regional or local scale for 10 out of the 32 national states and included a reduced number of causes and age groups. Therefore, we found an absence of national representative studies that describe the magnitude of blindness and vision impairment in the general population of Mexico.

**Added value of this study**

We used accurate and reliable data from the Global Burden of Disease study (GBD) 2019 to estimate the burden of blindness and vision impairment in Mexico during 2019. To the best of our knowledge, this study represents the first and most comprehensive description of the national burden of vision loss that includes severity and cause-specific visual impairment by sex and age. In addition, by using crude and age-standardized rates of years lived with disability, our analysis is intended to present a comparative assessment of the causes of vision loss that contribute the highest proportion of vision impairment.

**Implications of all the available evidence**

In Mexico, vision loss represents a public health problem that affects a great proportion of its population. The estimates presented herein might serve as a guide for developing population-based interventions aimed at reducing the burden of vision impairment, especially for avoidable blindness. In this regard, our results on the differential share of each cause to the severity of vision loss represent an opportunity for acting against those causes with the highest contribution of severe vision loss and blindness, particularly when visual impairment arises because of another disease. Given the complex geographic and socioeconomic features of Mexico, strong and concerted actions that highlight vision loss as a priority are needed to guarantee both high-quality health care and sustainable health management for the visually impaired population.

## Introduction

In 2019, the Global Burden of Disease (GBD) study estimated 1.2 billion prevalent cases of blindness and visual impairment for both sexes.^1^ During the same year, vision loss was ranked as the third global cause of impairment because it caused 26.5 million years lived with disability (YLDs), which accounted for 3.1% of the total global YLDs.^2^ Given the magnitude of the burden of vision loss, there is an ongoing effort to estimate both the prevalence and main causes of blindness and visual impairment. A previous GBD study estimated that 295 million people suffered moderate and severe vision impairment and 43.3 million were blind in 2020, with a greater proportion of cases in females.^3^ In addition, among adults older than 50 years, cataracts, glaucoma, uncorrected refractive errors, and diabetic retinopathy were the leading causes of blindness in 2020.^4^

With the aim of reducing avoidable visual impairment as a global public health issue, the 66th World Health Assembly set up a global target of reducing the prevalence of avoidable visual impairment by 25% by 2019.^5^ However, such a target was not reached because from 2010 to 2019, the global number of cases of avoidable blindness and moderate and severe vision impairment increased 10.8 and 31.5% in adults >50 years of age.^4^ Additionally, alarming increases in the number of cases across all four levels of severity of vision loss are projected by 2050, with the highest increase expected in uncorrected presbyopia, when passing from 510 million in 2020 to 866 million affected people in 2050.^3^

A concerted and stronger effort is required to reduce the global burden of vision loss. Therefore, focussing such effort in developing countries, where nearly 90% of visually impaired people live,^5^ may produce a significant impact toward the reduction of avoidable blindness and visual impairment, especially because 80% of vision loss can be prevented or cured. Consequently, it is necessary to design integrative national eye health plans that consider country priorities. However, in developing countries, little is known regarding how blindness and vision loss affect their population, and thus, their burden is underrated mainly because of the scarcity of studies and reports.

In Mexico, despite the research performed on describing the epidemiology of vision impairment, the studies are limited to some causes, years covered, regions, and age groups, which avoids a clear and broad understanding of the national burden of vision impairment. For instance, in Mexican children <7 years of age from urban areas of the centre of Mexico, retinopathy of prematurity, optic nerve atrophy, and congenital cataracts were the most frequent causes of blindness.^6^^,^^7^ In northern children between 12 and 13 years of age, myopia affected 40% of the subjects, although visual acuity was optimal, suggesting a late appearance of refractive disorders during adolescence.^8^ In adult patients >50 years of age from Northern Mexico, 1.7% of the population assessed was blind, with cataracts, diabetic retinopathy, and glaucoma being the main causes of blindness, while the prevalence of early, moderate and severe visual impairment ranged between 1.0 and 7.7%.^9^ In contrast, in the same population from the centre of Mexico, the prevalence of these three levels of vision impairment varied from 1.6 to 12.7%, with an estimated 1.0% of blind persons.^10^ In southern Mexico, 2.3% of the studied adults were blind, and a high prevalence of diabetic retinopathy was found.^11^ In southern Mexico, the prevalence of moderate visual impairment is higher in rural areas (10.2%) than in urban zones (3.9%).^12^

This heterogeneous body of evidence suggests the need for performing a national study that covers the general population and the leading causes of vision loss and presents this information by sex and age. Thus, the objective of this observational study is to present a demographically stratified comprehensive analysis of the burden of blindness and vision loss in Mexico during 2019 by severity and cause for each sex and age group using reliable and accurate data from the GBD 2019. These national estimates might help in understanding the magnitude and patterns of the vision loss burden and may serve as a guide for developing population-specific health policies aimed at alleviating vision loss.

## Methods

### Overview

We performed this observational study using results from the Global Burden of Disease study 2019 (GBD 2019). The GBD provides an updated comparative and descriptive epidemiology of health loss, which is based on a comprehensive and systematic assessment of 369 causes of diseases and injuries and their associated risk factors in 204 countries and territories from 1990 to 2019.^13^ The Methods Appendix 1 (pages 1418–1427) of the GBD 2019 diseases and injuries capstone study^1^ provides a detailed description of the approach and modelling steps used to estimate the blindness and vision loss impairment envelope as well as cause-specific vision loss. However, a brief description is presented in the following sections. The estimates included in this study were produced by the GBD in compliance with the Guidelines for Accurate and Transparent Health Estimates Reporting (GATHER) recommendations.^14^ This study was produced, supervised, and managed by members of the GBD Collaborative Network from Mexico and complies with the GBD protocol.

### Case definitions and cause-specific vision loss

The GBD study models visual acuity <6/18 according to the Snellen chart as a reference case, and according to the WHO criteria and International Statistical Classification of Diseases (ICD-10), it defines four levels of severity for blindness and vision loss: *near vision loss (presbyopia)*, near visual acuity of <6/12 distance equivalent; *moderate vision loss*, visual acuity ≥6/16 and < 6/18; *severe vision loss*, visual acuity ≥3/60 and < 6/60; and *blindness*, visual acuity of <3/60 or <10% visual field around central fixation.^3^

We included the following twelve causes of vision loss: near vision loss, uncorrected refractive error, glaucoma, cataract, diabetic retinopathy, age-related macular degeneration, trachoma, vitamin A deficiency, meningitis, encephalitis, retinopathy of prematurity (neonatal disorders), and a residual category of other causes of vision loss.^1^ The residual category “other causes of vision loss” includes all other causes of visual impairment not defined in the list. The case definitions for the 12 causes are presented in Supplementary Table S1 of the supplementary information.

Although GBD mapped onchocerciasis as a cause of vision impairment, we did not include it because this disease is eliminated from Mexico, and therefore, no data were retrieved for our study. Although trachoma was declared eliminated from Mexico in 2016,^15^ the data input sources used to estimate the burden of vision loss were published before this year. Consequently, the modelling approach used by the GBD still produces estimates for this disease. However, it is expected that with the inclusion of more recent data sources from Mexico, trachoma will be further eliminated as a cause of vision impairment in the country.

### Data input sources

To estimate the global, regional, and national burden of visual impairment, the GBD used mainly representative population-based surveys (peer-reviewed publications or gray literature) that report the measurement of visual acuity. Additionally, to estimate the prevalence of cause-specific vision loss, the GBD used a subset of the studies that reported vision impairment due to each cause. To find these data input sources, starting from GBD 2013 until the most recent iteration in 2019, the GBD conducted and updated several systematic reviews. In addition, for GBD 2016 and 2017, to find further data input sources, the GBD searched and extracted the Rapid Assessment Avoidable Blindness (RAAB) repository (https://www.raab.world/survey-data), which is a public database of studies reporting vision loss in developing countries. Finally, for GBD 2019, additional literature sources were provided by the Vision Loss Expert Group. Further details regarding model inputs are described on pages 1420–1422 of Methods Appendix 1 of GBD 2019.^1^ The Global Health Data Exchange (GHDx) Tool (https://ghdx.healthdata.org/gbd-2019/data-input-sources?components=5&impairments=200&locations=1) provides a detailed list and the citation of all the data sources. Globally, there were 481 data input sources used by the GBD to estimate the global burden of blindness and vision loss, among which five were from Mexico and included four research articles^9^, ^10^, ^11^^,^^16^ and one national survey.

### Modelling framework and estimation of severity- and cause-specific vision loss

For GBD 2019,^1^ the specific modelling framework that includes the flowchart and the computer coding for vision impairment estimation is available at GHDx (https://ghdx.healthdata.org/gbd-2019/code/nonfatal-13). Supplementary Table S2 provides links to the GHDx website that include the flowchart and codes of vision loss estimation due to each cause. The GBD study modelled vision loss in a two-step process: 1) severity-specific vision loss envelope estimation and 2) cause-specific vision loss estimation. Except for presbyopia (near vision loss), which was estimated directly during the first step, the prevalence of the other three levels of severity that cause distance vision loss and their causes was obtained from the second step.^1^

During the first step, four Disease Modelling Meta-Regression (DisMod-MR) 2.1 models were created to estimate the total prevalence of vision loss by severity (near vision loss, moderate vision loss, severe vision loss, and blindness) in conjunction with an additional model that estimates presenting vision loss (blindness + severe + moderate) that was used as covariate for improving consistency across the severity-specific models. In addition, a Bayesian meta-regression tool (MR-BRT)^17^ was used to split mixed severity data into severity-specific vision loss by adjusting a cubic spline on age. Furthermore, for each vision model, both the Healthcare Access and Quality Index and the Sociodemographic Index were used as covariates for each geographical location to indicate an indirect measure of access to eye care. During the second step, the prevalence of cause-specific vision loss was estimated for each cause mapped by the GBD, except for meningitis, neonatal disorders, and vitamin A deficiency, which were modelled as part of their underlying causes. Severity-specific models were produced with DisMod-MR 2.1 for each cause of vision loss, and for some causes, a combined model for moderate and severe vision loss was produced, whereas another single model was produced for blindness due to the cause.

### Location, age, and sex-specific estimates of vision loss

To estimate country-, age-, and sex-specific estimates, the GBD first produces global severity-specific and cause-specific estimates with a mixed effect nonlinear model that incorporates all the available data. Then, the outputs are fit to the seven GBD super regions and next to the GBD 21 regions before being finally fitted to the national and subnational levels.^1^ The final estimates were obtained by aggregation simply summing the corresponding level, from which the aggregated estimates were used to calculate sex specific, age specific, and age-standardized counts and rates per 100,000 people.^4^ For each case, the estimates were obtained from 1000 posterior draws of the model and calculating the mean across values of the draw level estimates along with 95% uncertainty intervals (UIs) that represent the 2.5th and 97.5th percentiles of the of the ordered draws.^3^

Data processing and analyses were conducted by the GBD using Python version 3.7.0 (Python Software Foundation); Stata version 15.1 (StataCorp); and R version 3.4.1 (R Foundation). The code is available at https://ghdx.healthdata.org/gbd-2019/code/nonfatal-13. We downloaded the estimates from the GHDx webpage as “csv” files to construct Excel databases and used Prism 9.5 (GraphPad, Inc. software) to construct all graphs and panels.

### Estimation of YLDs

The final estimates were used to calculate the years lived with disability (YLDs) due to vision impairment and blindness. For this, the GBD defined a series of disability weights (ranging from 0 “no health loss” to 1 “death”) caused by a specific sequelae disease that is multiplied by the prevalence of such disease.^18^ The severity-specific health state definitions and their disability weights provided by the GBD^1^ are summarized in Supplementary Table S3.

### Reporting standards

To estimate the burden of visual impairment and blindness in Mexico, we used data publicly available from the online GHDx query tool.^2^ We collected data at the national level by severity, cause, sex, and age during 2019. To summarize the burden, we used counts, crude, and age-standardized rates (ASR) per 100,000 people for the prevalence and YLDs during 2019.

### Role of the founding source

This study did not receive any funding.

## Results

### National burden of blindness and vision impairment by severity and cause in 2019

In Mexico during 2019, all the causes of blindness and vision loss mapped in the GBD and included in our study produced an estimated 11.01 million (95% UI, 9.25–13.11 million) prevalent cases of blindness and vision impairment and 384.96 thousand (259.57–544.25 thousand) YLDs for the general population (Table 1). After decomposing these figures according to the severity of vision impairment, 6.06 million (4.36–8.08 million) people were affected due to uncorrected presbyopia in Mexico, whereas moderate vision loss affected 3.81 million people (3.37–4.23 million). Additionally, there were 619.40 thousand (539.40–717.73 thousand) cases of severe vision loss, and 513.85 thousand (450.59–570.98) people were estimated to be blind in Mexico during 2019. With respect to YLDs counts, moderate vision loss and uncorrected presbyopia caused the highest and lowest burden, with 116.47 thousand (69.84–184.22 thousand) and 63.61 thousand (29.48–126.56 thousand) YLDs, respectively (Table 1).

**Table 1:** Estimates of crude and age-standardized (per 100,000 people) prevalence cases and YLDs counts of blindness and vision impairment by severity in Mexico for both sexes in 2019.

	Counts (95% UI)		ASR per 100,000 (95% UI)	
	Prevalence	YLDs	Prevalence	YLDs
**Category**				
Total	11,015,930 (9,256,915–13,110,851)	384,961 (259,571–544,249)	9410 (7896–11,230)	329 (222–464)
Presbyopia	6,065,377 (4,364,295–8,083,782)	63,611 (29,482–126,563)	5253 (3768–7011)	55 (25–109)
Moderate vision loss	3,817,296 (3,377,494–4,233,650)	116,466 (69,845–184,219)	3176 (2824–3515)	97 (58–153)
Severe vision loss	619,407 (539,400–717,731)	111,137 (74,298–160,846)	536 (466–623)	96 (64–139)
Blindness	513,849 (450,597–570,986)	93,748 (62,660–133,488)	446 (391–495)	81 (54–115)

YLDs, years-lived with disability; ASR, age-standardized rate; 95% UI, 95% uncertainty interval.

According to Table 1, the ASRs of prevalence and YLDs for total blindness and vision impairment were estimated at 9410 (7896–11,230) cases and 329 (222–464) YLDs counts per 100,000 people in 2019, respectively. In addition, among the four levels of severity of vision impairment, the ASR of prevalence ranged from 446 to 5253 cases per 100,000 people, with blindness and uncorrected presbyopia causing the lowest and highest burden, respectively. In contrast, with 97 (58–153) counts per 100,000 people, moderate vision loss caused the highest YLDs rate, and presbyopia caused the lowest (55, 25–109).

As summarized in Table 2, among the causes of blindness and vision loss that affected the general population of Mexico, near vision loss (6.06 million, 4.36–8.08) and refraction disorders (2.61 million, 2.3–2.93) were the two most prevalent. In contrast, with 3.72 thousand and 967 prevalent cases, encephalitis and meningitis, respectively, caused the lowest number of vision impairments. Regarding YLDs, refraction disorders were the main cause, with an estimated 120.43 thousand (80.68–170.71 thousand) counts, followed by cataracts, with 84.48 thousand (58.32–118.81 thousand). The ASR per 100,000 people ranged from 1 to 5253 prevalent cases and from 0.05 to 97 YLDs counts, with near vision loss causing the highest prevalence rate and the highest YLDs counts per 100,000 people attributed to refraction disorders (Table 2).

**Table 2:** Estimates of blindness and vision impairment in Mexico in 2019 for both sexes by cause.

	Counts (95% UI)		ASR per 100,000 (95% UI)	
	Prevalence	YLDs	Prevalence	YLDs
**Cause**				
Near vision loss	6,065,377 (4,364,295–8,083,782)	63,611 (29,482–126,563)	5253 (3768–7011)	55 (25–110)
Refraction disorders	2,613,822 (2,300,806–2,933,977)	120,428 (80,680–170,707)	2096 (1844–2346)	97 (66–139)
Cataract	1,117,414 (963,216–1,277,771)	84,485 (58,323–118,812)	1016 (876–1162)	77 (53–107)
Other causes of vision loss	583,825 (520,998–651,613)	52,698 (36,558–74,166)	504 (452–564)	45 (32–64)
Diabetic retinopathy	216,962 (174,297–268,246)	22,303 (14,422–31,762)	182 (147–226)	19 (12–26)
Glaucoma	131,840 (110,288–157,161)	13,107 (8806–18,522)	122 (102–146)	12 (8–17)
Vitamin A deficiency	105,370 (78,398–137,704)	7335 (4556–11,108)	87 (65–114)	6 (4–9)
Neonatal disorders	101,539 (81,699–122,597)	14,089 (9179–20,644)	80 (64–97)	11 (7–16)
Age-related macular degeneration	62,394 (51,382–73,982)	5757 (3832–8486)	57 (47–67)	5 (4–8)
Trachoma	12,701 (9487–16,569)	813 (521–1182)	10 (7–13)	0.64 (0.41–0.93)
Encephalitis	3719 (2947–4702)	267 (171–394)	3 (2–4)	0.21 (0.13–0.31)
Meningitis	967 (694–1314)	70 (40–109)	1 (0.55–1.04)	0.05 (0.03–0.08)

YLDs, years-lived with disability; ASR, age-standardized rate; 95% UI, 95% uncertainty interval.

### Sex-specific burden of blindness and vision impairment in 2019

**Fig. 1:**
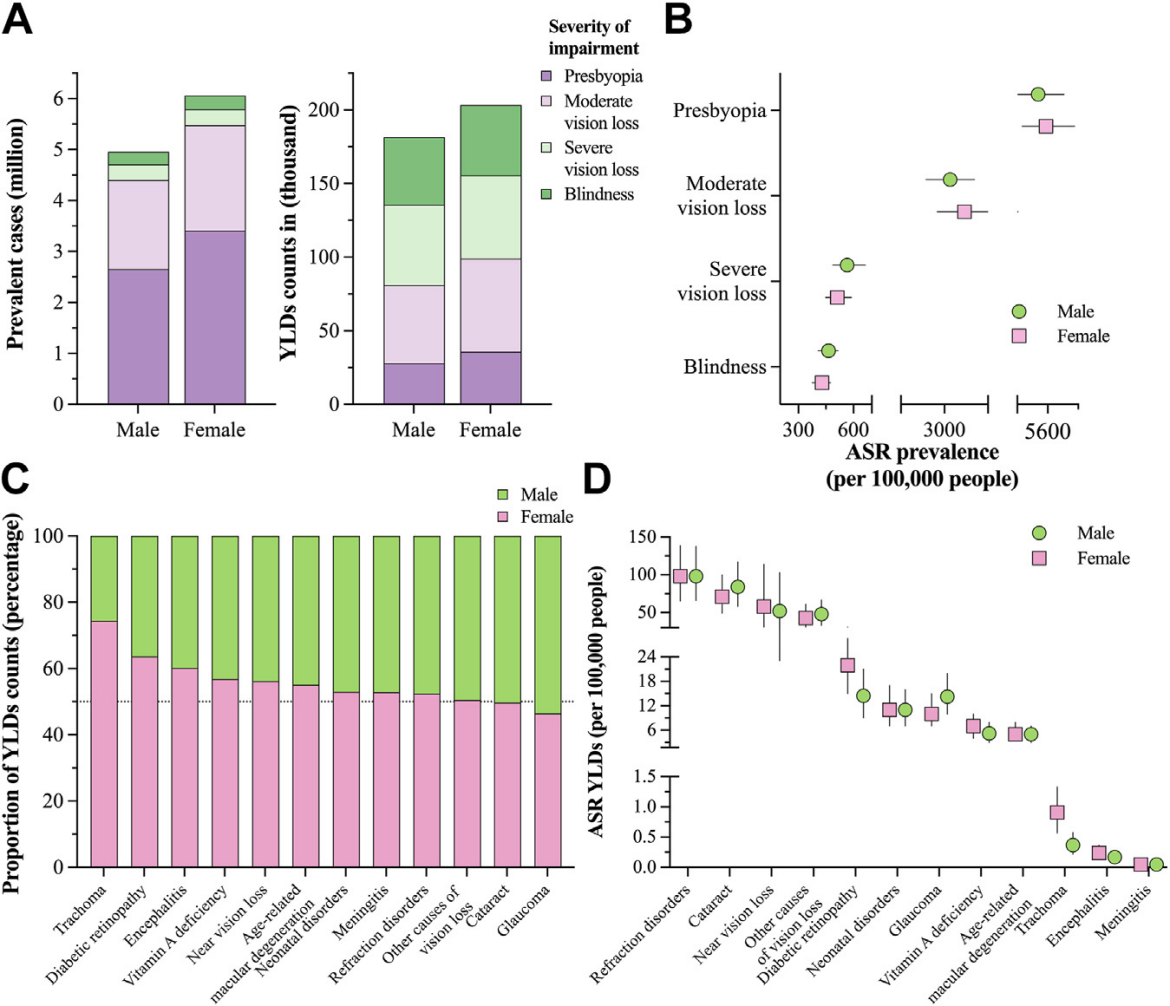
**Sex specific burden of blindness and vision loss in Mexico in 2019.** A) Prevalent cases and YLDs counts according to the severity of vision impairment by sex, B) sex-specific age-standardized rate of prevalence (per 100,000 people) by severity of vision impairment, C) proportion of YLDs counts for each cause by sex, and D) sex-specific age-standardized rate (per 100,000 people) of YLDs counts by cause.

Females of all ages suffered an overall higher burden of blindness and vision impairment in Mexico during 2019 (Fig. 1A). As summarized in Supplementary Table S4, of the total prevalent cases, females contributed 54.99% (6.05 million, 5.05–7.22) and 52.85% of the total YLDs counts (203.47 thousand, 136.60–287.69). According to the severity of the impairment, uncorrected presbyopia (3.40 million, 2.44–4.54) and moderate vision loss (2.07 million, 1.82–2.30) were more prevalent in females than males, as their estimations accounted for 56.18% and 54.27% of the total. In contrast, severe vision loss and blindness similarly affected both women and men, and similar trends were found for the YLDs (Supplementary Table S4). As depicted in Fig. 1B, females had a slightly higher ASR of prevalence (cases per 100,000 people) than males for uncorrected presbyopia (5495 vs. 4,973, respectively) and moderate vision loss (3276 vs. 3,079, respectively). However, this trend reverted as the ASR of prevalence for severe vision loss and blindness was higher in males than in females (Supplementary Table S5).

Except for glaucoma, the crude prevalence was higher in females than in males because females contributed between 52.07 and 81.69% of the total prevalent cases by cause (Supplementary Table S6). A similar pattern was found for YLDs, with trachoma and diabetic retinopathy showing the greatest gender disparity, as 74.41 and 63.64% of the YLDs due to these causes occurred in females. Indeed, females suffered a higher burden of YLDs counts in 9 out of 12 causes of vision loss in comparison to males (Fig. 1C). Regarding the ASR of prevalence per 100,000 people, females showed a higher rate in comparison to males in 8 out of 12 causes of vision impairment (Supplementary Table S7), whereas for the ASR of YLDs counts, cataract and glaucoma had higher estimates in males than females. In contrast, near vision loss, diabetic retinopathy, vitamin A deficiency, trachoma, and encephalitis caused a higher burden in females (Fig. 1D, Supplementary Table S7).

### Cause-specific contribution to the severity of blindness and vision impairment in 2019

**Fig. 2:**
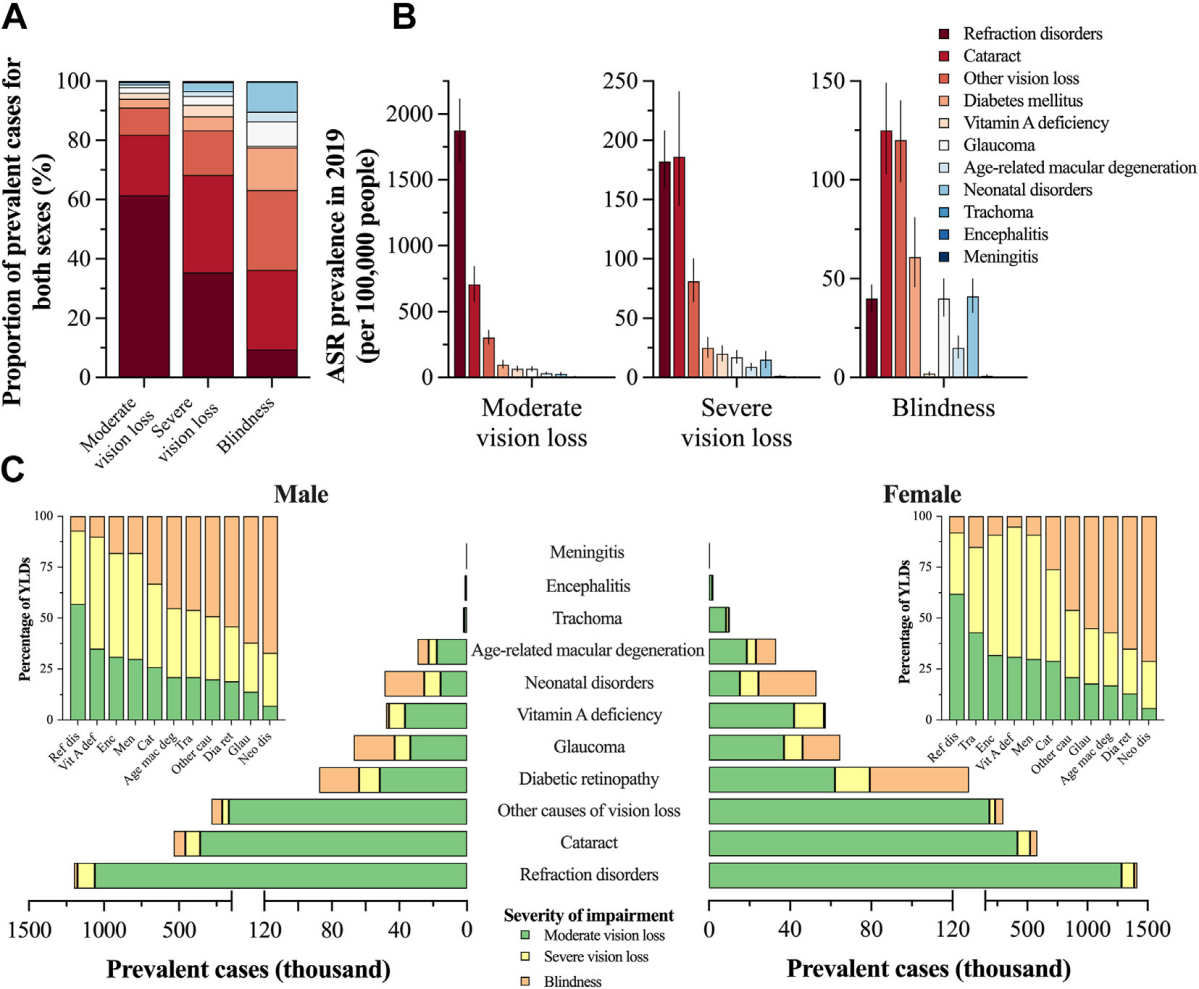
**Cause specific contribution according to the severity of vision impairment in Mexico in 2019.** A) Cause-specific relative contribution to the crude prevalence according to the severity of vision impairment, B) age-standardized rate of prevalence (per 100,000 people) by cause for each severity, and C) prevalent cases according to the severity of vision impairment by cause for each sex. In C, the insert shows the proportion of YLDs for each severity by cause.

Among the general population in Mexico, refraction disorders were the main cause of moderate (61.44% of total: 2.34 million cases, 2.04–2.64) and severe vision loss (35.43% of total: 219.50 thousand cases, 193.09–250.31) during 2019. In contrast, cataracts were the second most frequent cause of blindness (26.73% of the total), only behind the residual category other causes of vision loss (Fig. 2A, Supplementary Table S8). A similar trend was observed for YLD counts because refraction disorders were the leading cause of YLD counts among people affected with moderate and severe vision loss (Supplementary Table S9). As shown in Fig. 2B, the ASR of prevalence per 100,00 people for the three top causes of vision loss tended to decrease as the level of impairment increased (Supplementary Table S8). In contrast, the ASR of YLD counts per 100,000 people for diabetic retinopathy, glaucoma, neonatal disorders, age-related macular degeneration, and other causes of vision loss tended to increase according to the severity of the impairment (Supplementary Table S9).

According to Fig. 2C, the number of prevalent cases by severity for each cause of blindness and vision impairment showed an overall similar pattern between males and females from Mexico during 2019. Except for neonatal disorders, moderate vision loss was the most prevalent severity in all the causes of vision impairment; in males, moderate vision loss contributed 32.0–88.6% of the total prevalent cases for each cause, whereas in females, the percentage ranged from 28.7 to 90.6% (Supplementary Table S10). With respect to YLDs, the inserts of Fig. 2C revealed a contrasting pattern in the relative contribution of each level of severity by cause. Blindness was the leading cause of YLDs in neonatal disorders, diabetic retinopathy, age-related macular degeneration, glaucoma, and other causes of vision loss, whereas severe vision loss contributed to a higher percentage of YLDs in vitamin A deficiency, cataract, encephalitis, and meningitis.

### Age-specific burden of blindness and vision impairment in 2019

**Fig. 3:**
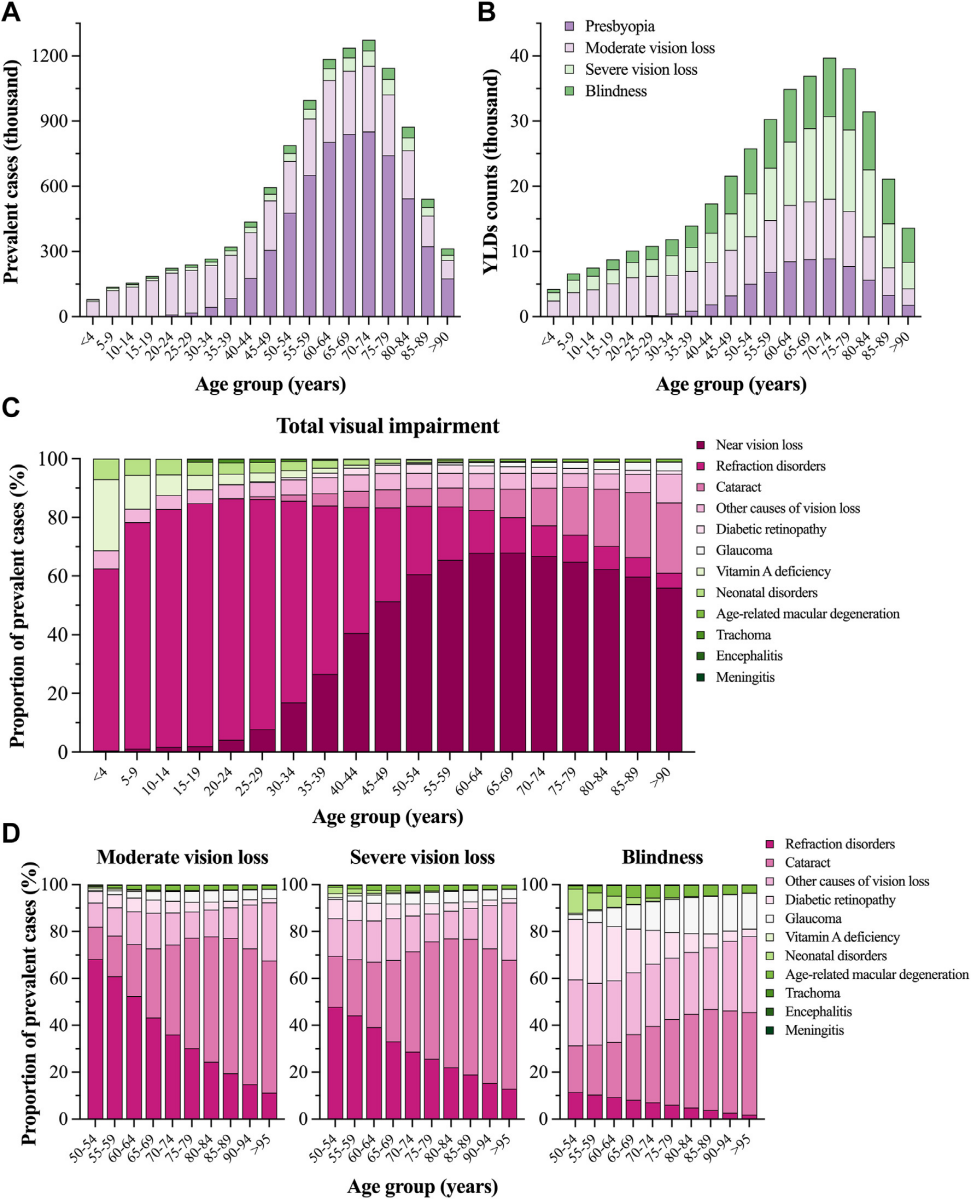
**Age specific burden of blindness and vision loss by cause and level of severity in Mexico in 2019.** Age-specific distribution of A) prevalent cases, B) YLDs counts according to the severity of vision impairment, C) cause-specific proportion of prevalent cases of total visual impairment for all age groups, and D) cause-specific proportion of prevalent cases according to the severity of impairment for adults >50 years of age.

According to Fig. 3, people >50 years of age suffered the highest burden of blindness and visual impairment in Mexico during 2019. With 1.27 million (9.76–1.64 million) prevalent cases, people of both sexes between 70 and 74 years of age were the most frequently visually impaired group (Supplementary Table S11). As depicted in Fig. 3A, the distribution of prevalent cases according to the severity of vision impairment showed that uncorrected presbyopia affected mainly the age groups starting from 45 to 49 years because it caused >50% of the cases in each group. In contrast, moderate vision loss caused 61.6–87.5% of the prevalent cases of vision impairment in younger age groups (<39 years), whereas severe vision loss and blindness were less frequent and affected almost consistently all age groups, as they contributed between 3.19 and 9.01% of the prevalent cases in each group (Supplementary Table S12). Like the prevalent cases, the distribution of YLD counts peaked in the 70–74 years age group (3.97 thousand, 26.56–57.06; Supplementary Table S11). However, as shown in Fig. 3B, there was a contrasting pattern regarding the burden of YLDs according to the severity of visual impairment. Especially in adults >50 years of age, severe vision loss and blindness were the two leading causes of YLDs because they contributed between 25.89 and 38.29% of all the counts in each age group (Supplementary Table S13).

As summarized in Fig. 3C, the cause-specific prevalence showed a distinctive pattern that varied according to the age group. Refractive errors, vitamin A deficiency, neonatal disorders, and other causes of vision loss in the residual group mainly affected the younger groups (<19 years of age). In contrast, near vision loss, cataract, diabetic retinopathy, and glaucoma became the leading causes of vision loss in adults >50 years of age. In addition, according to Fig. 3D, the relative contribution of the modelled causes of blindness and vision impairment for age groups older than 50 years showed a distinctive pattern that varied with the severity of the impairment. For both sexes, refraction disorders were the main contributors to moderate and severe vision loss in the age groups 50–54 to 65–69, although a consistent decrease was observed with age. In contrast, the percentage of prevalent cases due to cataracts increased consistently with age, becoming the leading cause of severe vision loss and blindness. Both diabetic retinopathy and neonatal disorders contributed mainly to the burden of blindness; however, they decreased with age, while glaucoma and age-related macular degeneration showed the opposite trend.

**Fig. 4:**
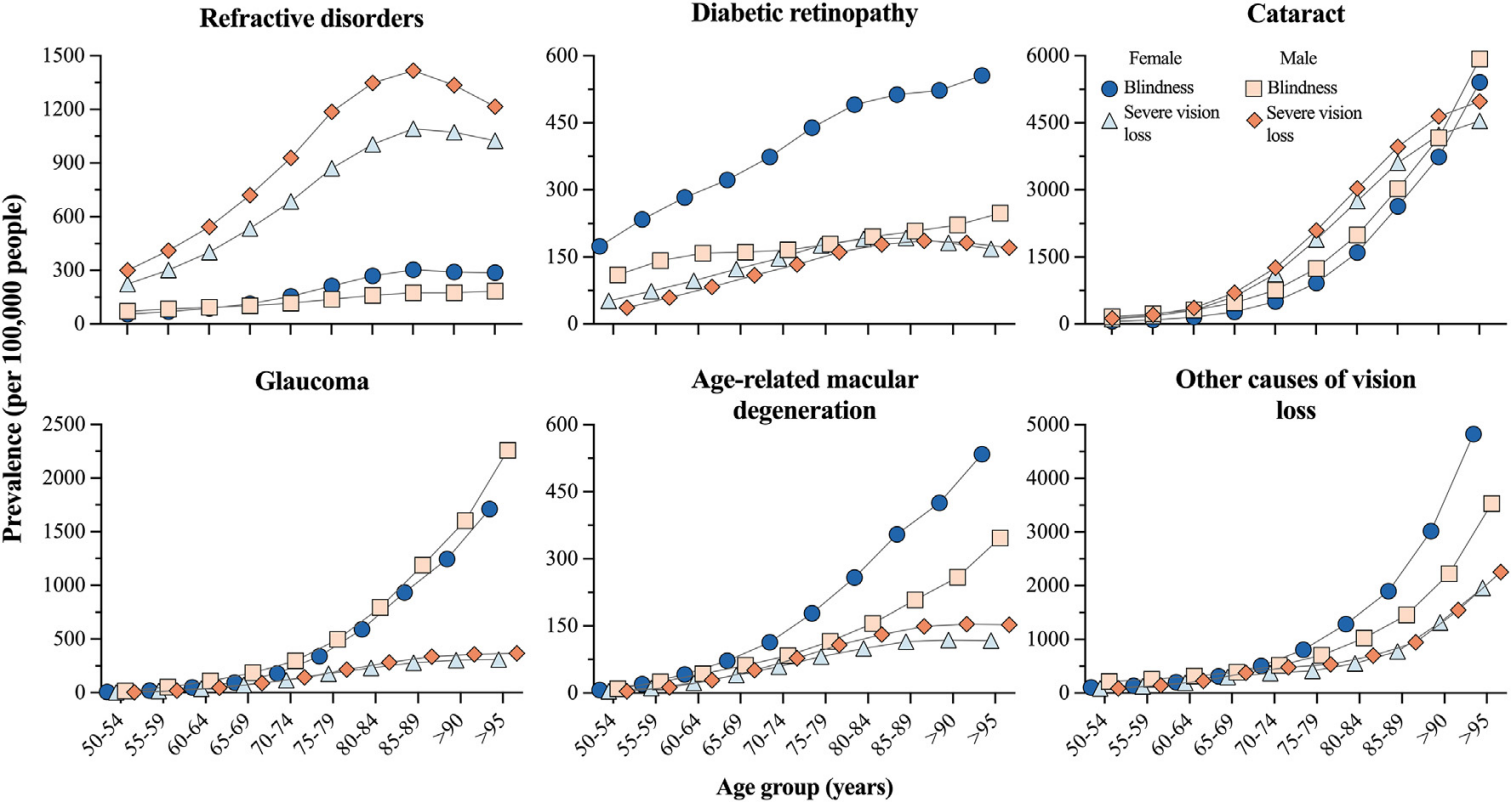
**Age and sex specific rate of prevalence by cause and level of severity of vision impairment in Mexico in 2019.** Age-specific prevalence rate (per 100,000 people) of severe vision loss and blindness in males and females for the six main causes of visual impairment.

Fig. 4 summarizes the sex-specific prevalence rates (per 100,000 people) for the six leading causes of severe vision loss and blindness in people older than 50 years of age (Supplementary Table S14). Except for cataracts, which increased with age and had similar prevalence rates between males and females, the remaining causes showed distinctive patterns for both gender and the severity of impairment. Refractive disorders caused a higher prevalence rate of severe vision loss in comparison to blindness, which additionally increased with age and caused a higher burden in males than in females. In contrast, glaucoma caused a higher burden of blindness, especially in age groups >75 years of age, but with similar estimations between sexes. Overall, diabetic retinopathy, age-related macular degeneration, and the residual group other causes of vision loss exhibited a similar pattern that consisted of an age-dependent increase and higher prevalence rates for blindness in females, especially in diabetic retinopathy.

## Discussion

The results presented herein suggest that in Mexico, blindness and vision impairment caused more than 11 million prevalent cases and 384 thousand YLDs. Given these figures, vision loss represents a public health problem with a crude prevalence of 9.35% (7.85–11.11%) in the general population during 2019. The high prevalence of vision loss found in Mexico is slightly below in comparison to the global crude prevalence of 11.05% and 9.85% for Central Latin America, which is a GBD region that comprises Mexico and five other countries, among which Costa Rica, Salvador, and Colombia had a higher burden of vision loss than Mexico.^2^ Nevertheless, the estimated value from Mexico is higher in comparison to countries from North America (Canada and the United States of America) and South America (Argentina, Chile, and Uruguay), whose prevalence ranged between 3.7 and 4.74%. These results show the existing heterogeneous and complex epidemiological pattern of blindness and vision impairment in the Americas. In addition, such disparity also indicates that most of the less developed countries from the continent suffer a greater burden of disease in comparison to developed ones.^19^ Consequently, blindness and vision impairment represent challenging public health issues in Mexico that require attention as well as increased public awareness in the prevention and treatment of vision loss.

With 55.06% of the prevalent cases, uncorrected presbyopia contributed the highest share of the visually impaired population in Mexico by 2019, which is a result consistent with previous global reports that showed that most of the cases of vision loss are attributed to near vision loss.^3^^,^^20^ Although uncorrected presbyopia remains the most common cause of visual impairment, it has mistakenly been perceived as less problematic than distance vision loss, especially in low- and middle-income countries, because of the lower life expectancy and reduced literacy.^21^ Nevertheless, regardless of the sociodemographic characteristics of a person, near vision loss is as detrimental to quality of life as the impairment of distance vision, and both conditions might interfere with the ability of the affected people to perform their activities of daily living.^22^ Therefore, to mitigate the high burden of vision loss caused by this easily treatable cause, there is an urgent need for a formal inclusion of near vision impairment by the ICD^21^ as well as population-based studies that help increase our understanding of the epidemiology and trends of uncorrected presbyopia. Our results also showed that uncorrected presbyopia was the leading cause of moderate and severe vision loss by 2019. Consequently, to reduce the high burden of vision loss caused by uncorrected presbyopia in low- and middle-income countries such as Mexico, a significant increase in the delivery of quality eye care by personnel trained in conjunction with affordable and sustainable spectacle delivery focused on the population at higher risk for developing permanent sight-threatening conditions is fundamental.^20^

Overall, our results showed a greater burden of vision loss in females than in males, which concurs with the global trends reported in previous studies.^23^^,^^24^ Some studies suggest that this tendency is caused by physiologic differences in accommodation, preferred reading distance, arm length, occupation, and light levels.^25^ Except for glaucoma, we found a higher number of prevalent cases in females than in males for the remaining causes of vision loss, which agrees with previous reports that highlight that women are more susceptible than men to developing these conditions.^26^ In addition to the specific causes of the distinct burden of vision loss between females and males, the existence of gender disparity has a significant impact on the ability of females to search for eye health-care programmes, mainly due to a different value in society, discriminatory distribution of resources, and unequal health-care seeking.^27^^,^^28^ Therefore, without first reducing the gender gap in Mexico and worldwide, females are expected to continue suffering a greater proportion of visual impairment. Indeed, a previous study concluded that the deficient analysis of inequality represents a problem in eye care and highlights the need for equity-relevant targets and indicators for eye health. Additionally, awareness and knowledge of eye conditions lead to patients seeking more frequent eye care monitoring.^29^

Among the people affected with blindness and vision impairment in Mexico during 2019, 40.27% of the prevalent cases were attributed to moderate and severe vision loss and 4.66% to blindness. Moderate and severe vision loss were the main contributors to YLDs in Mexico by 2019. Reversible causes such as refraction disorders and cataracts showed the highest age-standardized rate of YLDs per 100,000 people. In addition, among people affected with blindness in Mexico, cataracts, diabetic retinopathy, glaucoma, and refractive disorders showed the greatest age-standardized rates of prevalence per 100,000 people. Such a result contrasts with the ranking of causes of blindness in China, which excludes diabetic retinopathy and includes uncorrected refractive errors and age-related macular degeneration as the second and fourth leading causes.^30^ This difference may be because diabetic retinopathy is an emerging problem in Latin America because of the explosive increase in the number of type 2 diabetic patients, resulting from changes in dietary habits, sedentary lifestyles, and obesity.^31^ Globally, diabetic retinopathy affected 103.12 million people in 2020 and is projected to increase substantially during the following 30 years.^32^ Given that Mexico is among the countries with the highest prevalence of diabetes, such a trend for diabetic retinopathy suggests a deplorable eye health scenario for the Mexican population if no actions are implemented. Visual impairment due to diabetic retinopathy, as well as cataracts, glaucoma, and refraction disorders, is preventable; therefore, there is an urgency for developing or reinforcing national and regional eye health institutions that provide better assistance to prevent or treat these and other visual affections in the population.

Our results showed a distinct pattern of the burden of vision loss according to the age groups: moderate vision loss was most prevalent in people under 40 years, whereas presbyopia caused a higher burden in older groups. Among people between 50 and 69 years, refraction disorders were the main cause for moderate and severe vision loss, while cataracts caused the greatest cases of blindness in most of the age groups. Such results are similar to a recent global study, which showed that in adults older than 50 years, cataracts and glaucoma were the leading causes of blindness.^4^ The changing age structure of the population is causing a substantial increase in the number of cases of blindness and vision impairment.^19^ Therefore, in the Americas and several parts of the world, in conjunction with this rise in diabetes and obesity, population aging will increase vision impairment due to age-related eye diseases.^33^ Consequently, there is a need for increased public awareness regarding the effect of age on eye health care, especially in people suffering from diseases that threaten visual function.

This study is not devoid of several limitations and therefore some estimations should be interpreted with caution. First, there was a reduced number of data input sources for Mexico that may have increased the uncertainty of the estimations. Second, the lack of nationwide studies might affect the representativeness and understanding of the epidemiology of vision loss in the general population of Mexico. Third, although GBD used only representative population-based studies for the modelling of the estimations, the complex geography and socioeconomic disparities that exist in the country may induce bias regarding such representativeness, for instance, the population from rural and urban areas, or even people with native languages or that do not have access to health care facilities. Fourth, the disparity of definitions of blindness and vision impairment across the studies used as input sources in conjunction with the lack of a reporting standard may increase the uncertainty during the modelling. Sixth, the study only presents national estimates for 2019, which may cause an oversimplification of the true burden of vision loss at the subnational level and avoids a comparison of the trends. However, this last point warrants future subnational studies.

In conclusion, our results allowed us to accomplish the objective of the study, which consisted of describing the sex- and age-specific burden of blindness and vision loss by cause in Mexico during 2019. Our results demonstrated that vision impairment represents a public health problem affecting 11 million people in the territory. We found a gender disparity because females suffered a higher burden of YLDs in 9 out of 12 causes and exhibited higher prevalence rates of blindness and severe vision loss than males. In addition, our study revealed a complex age-specific pattern of blindness and vision loss in Mexico. In younger groups, we reported a higher proportion of vision loss due to refractive errors, neonatal disorders, and vitamin A deficiency, whereas near vision loss, cataracts, glaucoma, and diabetic retinopathy characterized vision impairment in adults >50 years of age. The results summarised herein contribute to the understanding and characterisation of a latent health problem among the population from Mexico.

Altogether, our study might provide valuable information since it compiles relevant data on how vision impairment causes a health burden in the population, mostly due to preventable causes. Since blindness and vision impairment represent challenging public health issues in Mexico, this study could support national health care decisions aimed at alleviating the vision loss burden among the most vulnerable populations within Mexico and in surrounding Central Latin American countries, which share ethnic and genetic similarities. However, it is fundamental to assess the prevalence of eye diseases and their impact on vision loss in each country. Therefore, the present study could guide further research in different settings or even at the subnational level in countries where there exists both great geographical heterogeneity and socioeconomic disparities. Given that more than half of the national burden of vision loss is attributed to uncorrected presbyopia, there is an opportunity to substantially reduce the magnitude of visual impairment in Mexico by developing and improving eye health-care systems that provide spectacles to the affected population. In addition, our study could also be used to enhance the distribution of eye care specialists (technicians, optometrists, and ophthalmologists) across the states of Mexico and incentivize the inclusion of eye care services within universal health care coverage. Finally, these results could aid in the development of public health policies aimed at preserving eye health and raise awareness of all causes of vision loss, including diseases such as diabetes and the effect of aging. These concerted actions could effectively induce a significant reduction in the burden of visual conditions that lead to avoidable blindness in Mexico and other countries from Latin America.

## Contributors

Conceptualization, SEMA, SABO, and DD; methodology, SABO, ELL, ELV, and PYGA; software, DZV, FJT, and PEHC; formal analysis, SABO, SLV, JMV, and DD; investigation, SEMA, PYGA, MR, FPM, NG<, and DD; data curation, DZV, SABO, and DD; writing—original draft preparation, SEMA, SABO, ELL, ELV, and JMMO; writing—review and editing, SABO and DD; visualization, DZV, FJT, and PEHC; supervision, JACG, ERG, and JMG; project administration, SABO, HCD, and DD. For this study, the corresponding and the leading authors had access to the data and decided to submit the study for publication.

## Data sharing statement

The datasets analyzed in the current study are available at the GHDx website (http://ghdx.healthdata.org/gbd-2019/data-input-sources).

## Declaration of interests

The authors declare that there are no conflicts of interest regarding the publication of this paper.

## Appendix A Supplementary data

Translate abstract
