# Supplementary material for: National sex- and age-specific burden of blindness and vision impairment by cause in Mexico in 2019: a secondary analysis of the Global Burden of Disease Study 2019
Source: Lancet Reg Health Am. 2023 Jul 11;24:100552. doi: 10.1016/j.lana.2023.100552 (PMC10339251; doi:10.1016/j.lana.2023.100552)
Supplement: Translate abstract [file mmc1.docx]

**Editor note:** *This translation in Spanish was submitted by the authors and we reproduce it as supplied. It has not been peer reviewed. Our editorial processes have only been applied to the original abstract in English, which should serve as reference for this manuscript*

**Resumen**

**Antecedentes** Las estimaciones nacionales confiables de ceguera y discapacidad visual son fundamentales para evaluar su carga de la enfermedad y desarrollar políticas de salud pública. Sin embargo, no se dispone de un análisis exhaustivo para México. Por lo tanto, en el presente estudio describimos la carga nacional de ceguera y pérdida de la visión por causa y gravedad durante 2019.

**Métodos** Utilizando datos públicos del estudio Global de Carga de la Enfermedad 2019, en este estudio, presentamos la prevalencia nacional y los recuentos de años vividos con discapacidad (AVD) y las tasas crudas y estandarizadas por edad (por cada 100,000 personas) de ceguera y pérdida de la visión total, por gravedad y por causa específica con intervalos de incertidumbre del 95% (II) por sexo y grupo de edad.

**Resultados** En México, la carga de ceguera y discapacidad visual se estimó en 11-01 millones (95% II, 9·25 a 13·11) casos prevalentes y 384·96 mil (259·57 a 544·24) AVD durante 2019. La presbicia no corregida causó la mayor carga (6·06 millones de casos, 4·36 a 8·08), mientras que la pérdida de visión severa y la ceguera afectaron a 619·40 mil (539·40 a 717·73) y 513·84 mil (450·59 a 570·98) personas, respectivamente. La pérdida de visión de cerca y los trastornos de refracción causaron el 78·7% de los casos, mientras que los trastornos neonatales y la degeneración macular asociada a la edad fueron de los menos frecuentes. Los trastornos de refracción fueron la principal causa de pérdida de visión moderada y grave (61·44 y 35·43%) y las cataratas fueron la segunda causa más frecuente de ceguera (26·73%). En general, las mujeres sufrieron una mayor carga de ceguera y discapacidad visual (54·99% y 52·85% del total de casos y AVD) y las personas >50 años sufrieron la mayor carga, siendo las personas entre 70 y 74 años las más afectadas.

**Interpretación** La pérdida de visión representa un problema de salud pública en México, siendo las mujeres y las personas mayores las más afectadas. Aunque las causas de la pérdida de visión contribuyen de manera diferencial a la gravedad de la discapacidad visual, la mayor parte de ésta es evitable. En consecuencia, es necesario un esfuerzo concertado a diferentes niveles para aliviar esta carga.

**Financiamiento** Este estudio no recibió financiamiento.

**Palabras clave:** ceguera evitable, carga de morbilidad, salud pública, pérdida de visión
