# Supplementary material for: National sex- and age-specific burden of blindness and vision impairment by cause in Mexico in 2019: a secondary analysis of the Global Burden of Disease Study 2019
Source: Lancet Reg Health Am. 2023 Jul 11;24:100552. doi: 10.1016/j.lana.2023.100552 (PMC10339251; doi:10.1016/j.lana.2023.100552)
Supplement: Supplementary Tables S1–S14 [file mmc2.docx]

**Supplementary Material**

[Supplementary Table 1. Case definitions and codes for the 10^th^ version of the ICD. 2](#_Toc130343292)

[Supplementary Table 2. Link to the flowchart and the code for each cause of vision loss. 3](#_Toc130343293)

[Supplementary Table 3. Health state and disability weights defined by the GBD. ^1^ 3](#_Toc130343294)

[Supplementary Table 4. Estimates of blindness and visual impairment in Mexico in 2019 by severity and sex. 4](#_Toc130343295)

[Supplementary Table 5. Age-standardized rate of prevalence and YLDs (per 100,000 people) of total blindness and visual impairment by severity and sex in Mexico during 2019. 4](#_Toc130343296)

[Supplementary Table 6. Estimates of blindness and visual impairment in Mexico in 2019 by cause and sex. 5](#_Toc130343297)

[Supplementary Table 7. Age-standardized (per 100,000 people) rate of prevalence and YLDs of total blindness and visual impairment by cause and sex in Mexico during 2019. 6](#_Toc130343298)

[Supplementary Table 8. Number of cases and age-standardized prevalence of blindness and vision impairment for each severity by cause in Mexico during 2019. 7](#_Toc130343299)

[Supplementary Table 9. Number of cases and age-standardized YLDs of blindness and vision impairment for each severity by cause in Mexico during 2019. 8](#_Toc130343300)

[Supplementary Table 10. Sex-specific prevalence (95% UI) and relative contribution by severity for each cause of blindness and vision impairment in Mexico 2019. 9](#_Toc130343301)

[Supplementary Table 11. Age-specific crude prevalence and YLDs counts of blindness and vision impairment in Mexico during 2019. 11](#_Toc130343302)

[Supplementary Table 12. Age-specific crude prevalence by severity of blindness and vision impairment in Mexico during 2019. 12](#_Toc130343303)

[Supplementary Table 13. Age-specific YLDs counts by severity of blindness and vision impairment in Mexico during 2019. 13](#_Toc130343304)

[Supplementary Table 14. Sex-specific rate of prevalence per 100,000 people by severity of blindness and vision impairment for each cause in 2019. 14](#_Toc130343305)

# Supplementary Table 1. Case definitions and codes for the 10^th^ version of the ICD.

| **Cause** | **Definition** | **ICD 10th** |
| --- | --- | --- |
| Near vision loss (presbyopia) | Defined as the age-related progressive inability of an individual to focus objects at a near distance and impairs reading but can be corrected by using contact lenes, reading glasses, or refractive surgery | H52.4 |
| Uncorrected refractive error | Defined as the presence of blurry vision caused by the lens’s focusing inability that can be addressed as in the case of presbyopia | H49 – H52 |
| Glaucoma | Defined as a condition characterized by an increase in the intraocular pressure that can provoke further damage to the optic nerve | H40 – H42 |
| Cataract | Defined as an impairment of vision caused by the clouding of the eye’s lens as a consequence of protein buildup | H25 |
| Diabetic retinopathy | Defined as a damage to the retina due to blood vessels malfunction that cause blood leak into the retina causing scarring to the retina | H36 |
| Age-related macular degeneration | Defined as central vision loss caused by the gradual deterioration of the macula | H35.3 |
| Trachoma | Defined as the scratching of the cornea caused by the inversion of the eyelids and eyelashes due to a conjunctival bacterial infection that progressively cause scarring of the cornea | H13.1 |
| Neonatal disorders (retinopathy of prematurity) | Defined as an abnormal development of retinal blood vessels in the retina in premature babies | H35.1 |
| Vitamin A deficiency | Defined as an inadequate intake of vitamin A | H19.8 |
| Meningitis | Defined as an infection of the surrounding membranes that protect the brain and the spinal cord | H48.1 |
| Encephalitis | Defined as an inflammation of the brain mainly caused by virus infections |  |
| Other vision loss | A residual group of any other cause of vision loss not defined in this list |  |

# Supplementary Table 2. Link to the flowchart and the code for each cause of vision loss.

| **Cause** | **GHDx code** |
| --- | --- |
| Cataract | <https://ghdx.healthdata.org/gbd-2019/code/nonfatal-2> |
| Diabetic retinopathy | <https://ghdx.healthdata.org/gbd-2019/code/nonfatal-3> |
| Encephalitis | <https://ghdx.healthdata.org/gbd-2019/code/nonfatal-4> |
| Glaucoma | <https://ghdx.healthdata.org/gbd-2019/code/nonfatal-5> |
| Macular degeneration | <https://ghdx.healthdata.org/gbd-2019/code/nonfatal-7> |
| Meningitis | <https://ghdx.healthdata.org/gbd-2019/code/nonfatal-8> |
| Neonatal disorders | <https://ghdx.healthdata.org/gbd-2019/code/nonfatal-9> |
| Other vision loss | <https://ghdx.healthdata.org/gbd-2019/code/nonfatal-10> |
| Refractive error | <https://ghdx.healthdata.org/gbd-2019/code/nonfatal-12> |
| Trachoma | <https://ghdx.healthdata.org/gbd-2019/code/nonfatal-12> |
| Vitamin A deficiency | <https://ghdx.healthdata.org/gbd-2019/code/nonfatal-13> |

# Supplementary Table 3. Health state and disability weights defined by the GBD. ^1^

| **Vision loss severity** | **Health state definition** | **Disability weight** |
| --- | --- | --- |
| Near vision loss | Difficult to see objects nearer than three feet in case that no reading glasses are used. However, the person experiences no difficult in seeing distant objects | 0.011 (0.005 to 0.02) |
| Distance vision, moderate loss | Difficult to recognize faces or objects distributed across a room | 0.031 (0.019 to 0.049) |
| Distance vision, severe loss | Due to severe vision loss, daily activities are difficult to complete. In consequence, there is an emotional impact due to a perceived difficulty for performing task or even going outside the home without proper assistance | 0.184 (0.125 to 0.259) |
| Distance vision blindness | The affected person is completely blind and has great difficulty in most of daily activities and task. This causes a great emotional impact and provokes a huge difficulty in going outside home without proper assistance | 0.187 (0.124 to 0.26) |

# Supplementary Table 4. Estimates of blindness and visual impairment in Mexico in 2019 by severity and sex.

| **Category** | **Prevalence counts (95% UI)** | | **Percentage of total by females** |  | **YLDs counts (95% UI)** | | **Percentage of total by females** |
| --- | --- | --- | --- | --- | --- | --- | --- |
|  | Female | Male |  |  | Female | Male |  |
| **TOTAL** | 6,057,965 (5,056,726 to 7,224,861) | 4,957,965 (4,166,743 to 5,874,479) | 54.99 |  | 203,468 (136,600 to 287,690) | 181,494 (123,512 to 255,225) | 52.85 |
| **Presbyopia** | 3,408,025 (2,441,785 to 4,549,975) | 2,657,351 (1,912,701 to 3,555,361) | 56.18 |  | 35,743 (16,587 to 71,008) | 27,868 (12,827 to 55,212) | 56.19 |
| **Moderate vision loss** | 2,071,641 (1,829,154 to 2,304,849) | 1,745,655 (1,546,287 to 1,936,999) | 54.27 |  | 63,203 (37,969 to 99,900) | 53,263 (31,921 to 84,316) | 54.27 |
| **Severe vision loss** | 315,725 (275,845 to 361,995) | 303,683 (262,915 to 353,977) | 50.97 |  | 56,670 (37,864 to 81,968) | 54,466 (36,482 to 78,805) | 50.10 |
| **Blindness** | 262,573 (230,370 to 290,982) | 251,276 (220,646 to 279,376) | 51.09 |  | 47,852 (31,974 to 67,911) | 45,897 (30,635 to 65,161) | 51.04 |

YLDs, years-lived with disability; 95% UI, 95% uncertainty interval

# Supplementary Table 5. Age-standardized rate of prevalence and YLDs (per 100,000 people) of total blindness and visual impairment by severity and sex in Mexico during 2019.

| **Category** | **ASR prevalence per 100,000 (95% UI)** | |  | **ASR YLDs per 100,000 (95% UI)** | |
| --- | --- | --- | --- | --- | --- |
|  | Female | Male |  | Female | Male |
| **TOTAL** | 9,712 (8,100 to 11,624) | 9,082 (7,643 to 10,809) |  | 327 (220 to 463) | 332 (226 to 466) |
| **Presbyopia** | 5,495 (3,925 to 7,366) | 4,973 (3,555 to 6,664) |  | 58 (27 to 114) | 52 (24 to 103) |
| **Moderate vision loss** | 3,276 (2,902 to 3,646) | 3,079 (2,748 to 3,416) |  | 100 (60 to 158) | 94 (56 to 148) |
| **Severe vision loss** | 512 (449 to 589) | 566 (487 to 665) |  | 92 (61 to 133) | 101 (67 to 147) |
| **Blindness** | 429 (376 to 476) | 464 (408 to 516) |  | 78 (52 to 111) | 84 (57 to 120) |

YLDs, years-lived with disability; ASR, age-standardized rate; 95% UI, 95% uncertainty interval

# Supplementary Table 6. Estimates of blindness and visual impairment in Mexico in 2019 by cause and sex.

| **Cause** | **Prevalence counts (95% UI)** | | **% of total by females** |  | **YLDs counts (95% UI)** | | **% of total by females** |
| --- | --- | --- | --- | --- | --- | --- | --- |
|  | Female | Male |  |  | Female | Male |  |
| **Near vision loss** | 3,408,025 (2,441,785 to 4,549,975) | 2,657,351 (1,912,701 to 3,555,361) | 56.18 |  | 35,743 (16,587 to 71,008) | 27,868 (12,827 to 55,212) | 56.19 |
| **Refraction disorders** | 1,415,557 (1,239,229 to 1,591,833) | 1,198,264 (1,057,211 to 1,339,506) | 54.16 |  | 63,169 (42,189 to 90,144) | 57,259 (38,476 to 80,710) | 52.45 |
| **Cataract** | 582,533 (498,777 to 668,993) | 534,881 (463,168 to 607,973) | 52.13 |  | 42,044 (29,056 to 59,410) | 42,441 (29,289 to 59,103) | 49.76 |
| **Other causes of vision loss** | 300,631 (268,613 to 337,086) | 283,194 (253,832 to 316,415) | 51.49 |  | 26,626 (18,465 to 37,737) | 26,072 (18,019 to 36,814) | 50.52 |
| **Diabetic retinopathy** | 129,660 (105,526 to 159,039) | 87,302 (69,570 to 110,267) | 59.76 |  | 14,194 (9,304 to 20,085) | 8,109 (5,205 to 11,614) | 63.64 |
| **Glaucoma** | 64,773 (53,849 to 77,905) | 67,067 (56,472 to 79,406) | 49.13 |  | 6,084 (4,082 to 8,594) | 7,023 (4,705 to 9,925) | 46.41 |
| **Vitamin A deficiency** | 57,626 (42,831 to 75,401) | 47,744 (35,424 to 62,443) | 54.69 |  | 4,168 (2,592 to 6,279) | 3,166 (1,952 to 4,826) | 56.83 |
| **Neonatal disorders** | 52,869 (43,321 to 63,928) | 48,670 (38,917 to 59,063) | 52.07 |  | 7,467 (4,862 to 10,905) | 6,622 (4,271 to 9,729) | 52.99 |
| **Age-related macular degeneration** | 33,153 (27,286 to 39,532) | 29,242 (23,950 to 34,692) | 53.13 |  | 3,170 (2,115 to 4,694) | 2,587 (1,712 to 3,797) | 55.06 |
| **Trachoma** | 10,376 (7,474 to 13,892) | 2,325 (1,751 to 3,025) | 81.69 |  | 605 (382 to 883) | 208 (125 to 323) | 74.41 |
| **Encephalitis** | 2,252 (1,756 to 2,883) | 1,467 (1,187 to 1,814) | 60.55 |  | 160 (101 to 242) | 106 (69 to 156) | 60.15 |
| **Meningitis** | 510 (362 to 698) | 457 (329 to 619) | 52.74 |  | 37 (21 to 58) | 33 (19 to 51) | 52.85 |

YLDs, years-lived with disability; 95% UI, 95% uncertainty interval

# Supplementary Table 7. Age-standardized (per 100,000 people) rate of prevalence and YLDs of total blindness and visual impairment by cause and sex in Mexico during 2019.

| **Cause** | **ASR prevalence per 100,000 (95% UI)** | |  | **ASR YLDs per 100,000 (95% UI)** | |
| --- | --- | --- | --- | --- | --- |
|  | Female | Male |  | Female | Male |
| **Near vision loss** | 5,495 (3,925 to 7,366) | 4,973 (3,555 to 6,664) |  | 58 (27 to 114) | 52 (24 to 103) |
| **Refraction disorders** | 2,184 (1,917 to 2,456) | 2,010 (1,784 to 2,246) |  | 98 (65 to 139) | 98 (66 to 138) |
| **Cataract** | 979 (839 to 1,125) | 1,059 (917 to 1,210) |  | 71 (49 to 100) | 84 (58 to 117) |
| **Other causes of vision loss** | 488 (436 to 546) | 523 (468 to 585) |  | 43 (30 to 61) | 48 (33 to 67) |
| **Diabetic retinopathy** | 204 (166 to 249) | 157 (125 to 199) |  | 22 (15 to 31) | 14 (9 to 21) |
| **Glaucoma** | 110 (92 to 132) | 136 (114 to 160) |  | 10 (7 to 15) | 14 (10 to 20) |
| **Vitamin A deficiency** | 94 (69 to 124) | 80 (59 to 104) |  | 7 (4 to 10) | 5 (3 to 8) |
| **Neonatal disorders** | 81 (67 to 99) | 79 (63 to 96) |  | 11 (7 to 17) | 11 (7 to 16) |
| **Age-related macular degeneration** | 56 (46 to 66) | 58 (47 to 68) |  | 5 (4 to 8) | 5 (3 to 7) |
| **Trachoma** | 16 (11 to 21) | 4 (3 to 5) |  | 0.91 (0.57 to 1.33) | 0.37 (0.22 to 0.58) |
| **Encephalitis** | 3 (3 to 4) | 2 (2 to 3) |  | 0.24 (0.15 to 0.37) | 0.17 (0.11 to 0.25) |
| **Meningitis** | 0.78 (0.55 to 1.07) | 0.75 (0.54 to 1.02) |  | 0.05 (0.03 to 0.08) | 0.06 (0.02 to 0.09) |

YLDs, years-lived with disability; ASR, age-standardized rate; 95% UI, 95% uncertainty interval

# Supplementary Table 8. Number of cases and age-standardized prevalence of blindness and vision impairment for each severity by cause in Mexico during 2019.

| **Cause** | **Moderate vision loss** | |  | **Severe vision loss** | |  | **Blindness** | |
| --- | --- | --- | --- | --- | --- | --- | --- | --- |
|  | Prevalence (95% UI) | ASR per 100,000 (95% UI) |  | Prevalence (95% UI) | ASR per 100,000 (95% UI) |  | Prevalence (95% UI) | ASR per 100,000 (95% UI) |
| **Refraction disorders** | 2,345,485 (2,042,554 to 2,649,328) | 1,874 (1,637 to 2,113) |  | 219,503 (193,091 to 250,311) | 182 (160 to 208) |  | 48,833 (41,198 to 57,426) | 40 (34 to 47) |
| **Cataract** | 776,701 (632,804 to 925,955) | 705 (575 to 839) |  | 203,346 (158,823 to 264,118) | 186 (145 to 241) |  | 137,367 (112,904 to 163,169) | 125 (103 to 149) |
| **Other causes of vision loss** | 351,955 (293,568 to 418,268) | 303 (254 to 359) |  | 93,480 (73,271 to 116,361) | 81 (64 to 100) |  | 138,390 (114,225 to 162,321) | 120 (99 to 140) |
| **Diabetic retinopathy** | 113,495 (81,976 to 154,335) | 96 (70 to 130) |  | 29,493 (20,441 to 40,758) | 25 (17 to 34) |  | 73,974 (55,763 to 98,105) | 61 (46 to 81) |
| **Glaucoma** | 70,532 (53,688 to 89,512) | 65 (49 to 82) |  | 18,463 (12,996 to 25,408) | 17 (12 to 23) |  | 42,845 (33,123 to 52,723) | 40 (31 to 50) |
| **Vitamin A deficiency** | 78,563 (55,019 to 106,183) | 65 (46 to 88) |  | 24,092 (17,308 to 33,246) | 20 (14 to 27) |  | 2,715 (1,444 to 4,263) | 2 (1 to 3) |
| **Neonatal disorders** | 30,797 (17,518 to 47,685) | 25 (14 to 38) |  | 18,767 (10,514 to 27,209) | 15 (8 to 22) |  | 51,975 (42,392 to 64,200) | 41 (33 to 50) |
| **Age-related macular degeneration** | 36,429 (28,046 to 45,757) | 33 (25 to 41) |  | 9,475 (6,803 to 12,725) | 9 (6 to 12) |  | 16,491 (11,326 to 22,619) | 15 (10 to 21) |
| **Trachoma** | 9,904 (6,853 to 13,624) | 8 (5 to 10) |  | 1,767 (1,300 to 2,353) | 1.37 (1.02 to 1.83) |  | 1,031 (660 to 1,520) | 0.90 (0.57 to 1.33) |
| **Encephalitis** | 2,730 (2,088 to 3,530) | 2 (2 to 3) |  | 810 (571 to 1,093) | 0.64 (0.45 to 0.86) |  | 179 (122 to 248) | 0.14 (0.09 to 0.19) |
| **Meningitis** | 706 (479 to 987) | 0.56 (0.38 to 0.77) |  | 211 (140 to 302) | 0.16 (0.11 to 0.23) |  | 49 (31 to 73) | 0.03 (0.02 to 0.05) |

ASR, age-standardized rate; 95% UI, 95% uncertainty interval

# Supplementary Table 9. Number of cases and age-standardized YLDs of blindness and vision impairment for each severity by cause in Mexico during 2019.

| **Cause** | **Moderate vision loss** | |  | **Severe vision loss** | |  | **Blindness** | |
| --- | --- | --- | --- | --- | --- | --- | --- | --- |
|  | YLDs (95% UI) | ASR per 100,000 (95% UI) |  | YLDs (95% UI) | ASR per 100,000 (95% UI) |  | YLDs (95% UI) | ASR per 100,000 (95% UI) |
| **Refraction disorders** | 71,857 (43,395 to 113,446) | 57 (35 to 91) |  | 39,596 (26,287 to 58,068) | 33 (22 to 48) |  | 8,975 (5,926 to 13,138) | 7 (5 to 11) |
| **Cataract** | 23,479 (13,482 to 37,318) | 21 (12 to 34) |  | 36,166 (22,687 to 54,175) | 33 (21 to 50) |  | 24,840 (16,398 to 36,842) | 23 (15 to 33) |
| **Other causes of vision loss** | 10,683 (6,310 to 16,942) | 9 (5 to 15) |  | 16,783 (10,660 to 25,169) | 14 (9 to 22) |  | 25,232 (16,655 to 36,375) | 22 (14 to 32) |
| **Diabetic retinopathy** | 3,455 (1,799 to 5,771) | 3 (2 to 5) |  | 5,290 (3,078 to 8,246) | 4 (3 to 7) |  | 13,558 (8,113 to 20,076) | 11 (7 to 17) |
| **Glaucoma** | 2,130 (1,195 to 3,452) | 2 (1 to 3) |  | 3,278 (1,914 to 5,261) | 3 (2 to 5) |  | 7,700 (4,784 to 11,381) | 7 (5 to 11) |
| **Vitamin A deficiency** | 2,413 (1,292 to 4,121) | 2 (1 to 3) |  | 4,413 (2,600 to 6,900) | 4 (2 to 6) |  | 509 (249 to 882) | 0.40 (0.19 to 0.69) |
| **Neonatal disorders** | 940 (455 to 1,704) | 0.75 (0.36 to 1.36) |  | 3,417 (1,712 to 5,782) | 3 (1 to 5) |  | 9,732 (6,211 to 14,425) | 8 (5 to 11) |
| **Age-related macular degeneration** | 1,099 (610 to 1,737) | 0.99 (0.54 to 1.57) |  | 1,685 (1,021 to 2,609) | 1.52 (0.92 to 2.35) |  | 2,973 (1,815 to 4,656) | 3 (2 to 4) |
| **Trachoma** | 304 (163 to 508) | 0.23 (0.12 to 0.38) |  | 322 (181 to 512) | 0.25 (0.14 to 0.39) |  | 187 (96 to 325) | 0.16 (0.08 to 0.28) |
| **Encephalitis** | 84 (48 to 137) | 0.06 (0.03 to 0.11) |  | 149 (88 to 233) | 0.11 (0.07 to 0.18) |  | 33 (20 to 53) | 0.02 (0.01 to 0.04) |
| **Meningitis** | 22 (11 to 38) | 0.02 (0.01 to 0.03) |  | 39 (21 to 65) | 0.03 (0.02 to 0.05) |  | 9 (5 to 15) | 0.01 (0.0 to 0.01) |

YLDs, years-lived with disability; ASR, age-standardized rate; 95% UI, 95% uncertainty interval

# Supplementary Table 10. Sex-specific prevalence (95% UI) and relative contribution by severity for each cause of blindness and vision impairment in Mexico 2019.

| **Cause** | **Moderate vision loss** | | **Severe vision loss** | | **Blindness** | |
| --- | --- | --- | --- | --- | --- | --- |
|  | Female | Percentage | Female | Percentage | Female | Percentage |
| **Refraction disorders** | 1,283,802 (1,113,330 to 1,454,799) | 90.69 | 105,375 (92,443 to 120,380) | 7.44 | 26,379 (22,134 to 30,957) | 1.86 |
| **Cataract** | 417,992 (340,150 to 498,649) | 71.75 | 105,146 (82,306 to 135,745) | 18.05 | 59,394 (47,761 to 71,892) | 10.19 |
| **Other causes of vision loss** | 184,404 (154,068 to 218,238) | 61.33 | 48,408 (38,288 to 59,642) | 16.10 | 67,820 (55,667 to 80,569) | 22.56 |
| **Diabetic retinopathy** | 62,145 (44,970 to 83,773) | 47.92 | 17,230 (12,125 to 23,571) | 13.28 | 50,285 (38,583 to 65,558) | 38.78 |
| **Glaucoma** | 37,001 (28,249 to 47,177) | 57.12 | 9,161 (6,479 to 12,619) | 14.14 | 18,611 (13,908 to 23,143) | 28.73 |
| **Vitamin A deficiency** | 41,951 (29,347 to 56,585) | 72.79 | 14,645 (10,437 to 20,275) | 25.41 | 1,030 (567 to 1,599) | 1.78 |
| **Neonatal disorders** | 15,208 (8,687 to 23,679) | 28.76 | 9,185 (4,979 to 13,562) | 17.37 | 28,476 (23,135 to 35,169) | 53.86 |
| **Age-related macular degeneration** | 18,620 (14,366 to 23,553) | 56.16 | 4,557 (3,293 to 6,116) | 13.74 | 9,977 (6,855 to 13,853) | 30.09 |
| **Trachoma** | 8,488 (5,733 to 11,772) | 81.80 | 1,381 (1,023 to 1,854) | 13.31 | 507 (320 to 760) | 4.88 |
| **Encephalitis** | 1,659 (1,253 to 2,178) | 73.66 | 516 (355 to 703) | 22.91 | 77 (52 to 108) | 3.41 |
| **Meningitis** | 372 (251 to 521) | 72.94 | 120 (79 to 174) | 23.52 | 18 (12 to 27) | 3.52 |
|  | Male | Percentage | Male | Percentage | Male | Percentage |
| **Refraction disorders** | 1,061,682 (927,376 to 1,196,251) | 88.60 | 114,128 (100,120 to 130,529) | 9.52 | 22,454 (18,741 to 26,380) | 1.87 |
| **Cataract** | 358,708 (293,000 to 427,540) | 67.06 | 98,200 (76,105 to 128,994) | 18.35 | 77,973 (64,614 to 92,167) | 14.57 |
| **Other causes of vision loss** | 167,552 (139,736 to 200,363) | 59.16 | 45,072 (34,955 to 56,721) | 15.91 | 70,570 (58,606 to 82,621) | 24.91 |
| **Diabetic retinopathy** | 51,350 (37,030 to 70,005) | 58.90 | 12,262 (8,321 to 17,062) | 14.01 | 23,689 (17,036 to 33,226) | 27.07 |
| **Glaucoma** | 33,532 (25,522 to 42,520) | 49.99 | 9,302 (6,525 to 12,795) | 13.87 | 24,234 (18,951 to 29,517) | 36.13 |
| **Vitamin A deficiency** | 36,612 (25,879 to 49,251) | 76.68 | 9,447 (6,669 to 13,008) | 19.78 | 1,685 (889 to 2,618) | 3.52 |
| **Neonatal disorders** | 15,589 (8,686 to 24,188) | 32.03 | 9,581 (5,473 to 13,862) | 19.68 | 23,500 (19,045 to 29,182) | 48.28 |
| **Age-related macular degeneration** | 17,809 (13,767 to 22,403) | 60.90 | 4,918 (3,498 to 6,641) | 16.81 | 6,515 (4,487 to 8,823) | 22.28 |
| **Trachoma** | 1,416 (958 to 1,994) | 60.90 | 385 (262 to 545) | 16.56 | 524 (324 to 781) | 22.54 |
| **Encephalitis** | 1,071 (827 to 1,350) | 73.00 | 294 (212 to 389) | 20.04 | 102 (71 to 142) | 6.95 |
| **Meningitis** | 334 (231 to 464) | 73.08 | 92 (61 to 130) | 20.13 | 31 (20 to 46) | 6.78 |

95% UI, 95% uncertainty interval

# Supplementary Table 11. Age-specific crude prevalence and YLDs counts of blindness and vision impairment in Mexico during 2019.

| **Age group** | **Prevalent cases (95% UI)** | **YLDs counts (95% UI)** |  |
| --- | --- | --- | --- |
| **<1** | 9,295 (6,691 to 13,056) | 466 (286 to 712) |  |
| **1-4** | 80,116 (61,962 to 102,087) | 3,785 (2,448 to 5,570) |  |
| **5-9** | 137,822 (104,960 to 175,745) | 6,598 (4,255 to 9,615) |  |
| **10-14** | 155,737 (118,989 to 202,225) | 7,522 (4,866 to 11,158) |  |
| **15-19** | 187,150 (146,758 to 236,540) | 8,767 (5,614 to 12,748) |  |
| **20-24** | 224,312 (169,910 to 291,980) | 10,106 (6,396 to 14,830) |  |
| **25-29** | 239,684 (188,467 to 292,729) | 10,827 (7,071 to 15,546) |  |
| **30-34** | 267,058 (209,807 to 325,713) | 11,865 (7,755 to 17,387) |  |
| **35-39** | 321,855 (258,156 to 402,623) | 13,958 (9,194 to 20,006) |  |
| **40-44** | 437,348 (342,597 to 562,220) | 17,356 (11,429 to 25,157) |  |
| **45-49** | 596,500 (455,653 to 767,671) | 21,629 (14,506 to 31,924) |  |
| **50-54** | 789,523 (593,581 to 1,012,677) | 25,812 (17,054 to 38,278) |  |
| **55-59** | 996,895 (728,489 to 1,318,747) | 30,324 (20,392 to 45,318) |  |
| **60-64** | 1,186,175 (900,967 to 1,571,251) | 34,917 (23,411 to 51,657) |  |
| **65-69** | 1,237,147 (916,562 to 1,641,305) | 36,931 (24,475 to 53,719) |  |
| **70-74** | 1,274,399 (976,552 to 1,645,109) | 39,718 (26,563 to 57,062) |  |
| **75-79** | 1,145,224 (878,773 to 1,535,287) | 38,095 (25,894 to 54,963) |  |
| **80-84** | 874,182 (676,305 to 1,154,824) | 31,503 (21,445 to 44,701) |  |
| **85-89** | 542,169 (416,922 to 691,987) | 21,163 (14,542 to 29,339) |  |
| **90-94** | 236,232 (181,456 to 297,166) | 10,005 (6,810 to 13,823) |  |
| **>95** | 77,108 (57,864 to 97,784) | 3,613 (2,478 to 4,983) |  |

YLDs, years-lived with disability; 95% UI, 95% uncertainty interval

# Supplementary Table 12. Age-specific crude prevalence by severity of blindness and vision impairment in Mexico during 2019.

| **Age group** | **Prevalence counts (95% UI)** | | | |
| --- | --- | --- | --- | --- |
|  | Presbyopia | Moderate vision loss | Severe vision loss | Blindness |
| **<1** | 17 (8 to 32) | 8,099 (5,550 to 11,945) | 916 (609 to 1,575) | 263 (114 to 459) |
| **1-4** | 484 (221 to 920) | 70,992 (53,036 to 93,135) | 6,332 (4,967 to 8,005) | 2,308 (1,477 to 3,296) |
| **5-9** | 1,590 (726 to 3,023) | 120,669 (88,193 to 158,664) | 10,602 (8,205 to 13,491) | 4,961 (3,381 to 6,759) |
| **10-14** | 2,710 (1,237 to 5,154) | 134,946 (98,532 to 181,962) | 11,420 (8,555 to 14,891) | 6,661 (4,922 to 8,796) |
| **15-19** | 3,778 (1,725 to 7,185) | 163,366 (124,101 to 212,511) | 11,964 (8,745 to 16,291) | 8,042 (6,001 to 10,389) |
| **20-24** | 9,357 (4,713 to 16,020) | 192,896 (137,253 to 260,843) | 12,767 (9,160 to 17,770) | 9,292 (7,157 to 11,779) |
| **25-29** | 18,527 (9,512 to 32,755) | 196,301 (147,209 to 246,897) | 14,139 (10,623 to 18,895) | 10,717 (8,460 to 13,280) |
| **30-34** | 45,055 (25,272 to 74,609) | 192,420 (141,323 to 247,364) | 16,268 (11,965 to 21,753) | 13,316 (10,595 to 16,263) |
| **35-39** | 85,679 (47,281 to 146,307) | 198,543 (148,876 to 250,008) | 19,906 (14,925 to 25,259) | 17,727 (14,061 to 21,622) |
| **40-44** | 177,552 (109,060 to 272,589) | 210,984 (155,420 to 282,543) | 24,615 (18,580 to 31,393) | 24,197 (18,923 to 29,549) |
| **45-49** | 306,569 (181,699 to 472,899) | 228,129 (171,224 to 292,254) | 30,705 (23,925 to 38,583) | 31,097 (25,052 to 39,286) |
| **50-54** | 478,317 (297,249 to 691,500) | 237,896 (175,296 to 313,969) | 36,217 (27,960 to 46,958) | 37,092 (29,252 to 45,548) |
| **55-59** | 652,556 (382,772 to 975,603) | 259,561 (201,948 to 337,261) | 44,208 (34,269 to 56,183) | 40,570 (32,827 to 48,973) |
| **60-64** | 804,583 (505,121 to 1,166,955) | 284,011 (216,736 to 379,065) | 53,798 (41,397 to 68,534) | 43,783 (36,229 to 52,575) |
| **65-69** | 840,182 (530,663 to 1,224,002) | 290,661 (223,682 to 369,023) | 62,446 (46,697 to 82,225) | 43,859 (35,528 to 53,295) |
| **70-74** | 851,841 (562,059 to 1,224,661) | 302,187 (231,256 to 390,557) | 71,163 (52,315 to 93,340) | 49,208 (39,790 to 59,664) |
| **75-79** | 742,644 (483,791 to 1,109,558) | 279,789 (217,000 to 350,633) | 70,605 (53,714 to 94,708) | 52,186 (41,407 to 64,167) |
| **80-84** | 544,836 (357,328 to 806,802) | 220,774 (166,270 to 285,681) | 58,679 (44,270 to 79,853) | 49,893 (39,961 to 61,322) |
| **85-89** | 323,670 (209,945 to 467,527) | 140,745 (112,259 to 172,308) | 38,875 (29,879 to 51,006) | 38,880 (31,310 to 47,514) |
| **90-94** | 134,342 (83,251 to 192,720) | 63,135 (50,606 to 78,614) | 17,847 (13,072 to 24,017) | 20,907 (16,954 to 25,909) |
| **>95** | 41,089 (22,550 to 59,521) | 21,193 (16,429 to 26,792) | 5,937 (4,126 to 8,167) | 8,889 (7,231 to 11,088) |

95% UI, 95% uncertainty interval

# Supplementary Table 13. Age-specific YLDs counts by severity of blindness and vision impairment in Mexico during 2019.

| **Age group** | **YLDs counts (95% UI)** | | | |
| --- | --- | --- | --- | --- |
|  | Presbyopia | Moderate vision loss | Severe vision loss | Blindness |
| **<1** | 0 (0 to 0) | 249 (133 to 436) | 168 (90 to 303) | 49 (20 to 93) |
| **1-4** | 5 (2 to 13) | 2,186 (1,236 to 3,681) | 1,161 (749 to 1,732) | 432 (231 to 724) |
| **5-9** | 17 (5 to 42) | 3,718 (2,030 to 6,151) | 1,934 (1,219 to 2,930) | 929 (530 to 1,460) |
| **10-14** | 29 (9 to 70) | 4,159 (2,401 to 7,030) | 2,089 (1,278 to 3,194) | 1,244 (738 to 1,898) |
| **15-19** | 40 (13 to 98) | 5,031 (2,798 to 8,341) | 2,192 (1,336 to 3,408) | 1,503 (937 to 2,264) |
| **20-24** | 99 (36 to 220) | 5,936 (3,209 to 9,754) | 2,335 (1,372 to 3,615) | 1,736 (1,081 to 2,623) |
| **25-29** | 196 (72 to 447) | 6,041 (3,368 to 9,595) | 2,589 (1,593 to 4,034) | 2,001 (1,263 to 2,982) |
| **30-34** | 476 (178 to 1,045) | 5,918 (3,199 to 9,569) | 2,984 (1,797 to 4,636) | 2,487 (1,544 to 3,711) |
| **35-39** | 904 (328 to 1,981) | 6,107 (3,402 to 9,795) | 3,642 (2,176 to 5,444) | 3,304 (2,064 to 4,873) |
| **40-44** | 1,877 (748 to 4,125) | 6,485 (3,539 to 10,892) | 4,490 (2,778 to 6,725) | 4,503 (2,857 to 6,711) |
| **45-49** | 3,244 (1,263 to 7,058) | 7,003 (4,018 to 11,520) | 5,600 (3,503 to 8,406) | 5,783 (3,586 to 8,577) |
| **50-54** | 5,050 (2,014 to 10,824) | 7,289 (4,116 to 12,116) | 6,594 (4,106 to 10,042) | 6,879 (4,338 to 10,290) |
| **55-59** | 6,874 (2,676 to 14,809) | 7,931 (4,405 to 12,994) | 8,024 (4,921 to 12,105) | 7,495 (4,873 to 10,923) |
| **60-64** | 8,467 (3,581 to 18,246) | 8,655 (4,749 to 13,959) | 9,734 (5,842 to 14,719) | 8,061 (5,213 to 11,800) |
| **65-69** | 8,835 (3,606 to 18,236) | 8,833 (4,931 to 14,322) | 11,238 (7,043 to 17,334) | 8,026 (5,231 to 11,938) |
| **70-74** | 8,931 (3,761 to 17,995) | 9,147 (5,052 to 14,921) | 12,707 (7,939 to 19,669) | 8,932 (5,893 to 13,377) |
| **75-79** | 7,756 (3,224 to 15,799) | 8,436 (4,813 to 13,397) | 12,505 (7,745 to 19,243) | 9,398 (6,099 to 14,164) |
| **80-84** | 5,664 (2,389 to 11,486) | 6,630 (3,708 to 10,842) | 10,301 (6,431 to 16,137) | 8,908 (5,680 to 13,287) |
| **85-89** | 3,343 (1,450 to 6,365) | 4,205 (2,409 to 6,819) | 6,754 (4,179 to 10,197) | 6,862 (4,416 to 10,033) |
| **90-94** | 1,382 (583 to 2,661) | 1,879 (1,084 to 3,055) | 3,078 (1,888 to 4,806) | 3,666 (2,374 to 5,378) |
| **>95** | 421 (169 to 852) | 627 (349 to 1,030) | 1,016 (593 to 1,546) | 1,549 (993 to 2,263) |

YLDs, years-lived with disability; 95% UI, 95% uncertainty interval

# Supplementary Table 14. Sex-specific rate of prevalence per 100,000 people by severity of blindness and vision impairment for each cause in 2019.

| **Age group** | **Prevalence per 100,000 (95% UI)** | | | |
| --- | --- | --- | --- | --- |
|  | **Female** | | **Male** | |
|  | Severe vision loss | Blindness | Severe vision loss | Blindness |
| **Refractive disorders** | | | | |
| **50-54** | 225 (169 to 293) | 56 (39 to 77) | 301 (229 to 389) | 72 (50 to 99) |
| **55-59** | 303 (229 to 385) | 70 (49 to 92) | 411 (313 to 518) | 85 (60 to 112) |
| **60-64** | 403 (303 to 520) | 88 (63 to 119) | 543 (410 to 700) | 94 (69 to 125) |
| **65-69** | 535 (398 to 703) | 114 (79 to 156) | 720 (538 to 944) | 102 (72 to 136) |
| **70-74** | 686 (501 to 917) | 155 (109 to 211) | 929 (684 to 1,245) | 117 (82 to 159) |
| **75-79** | 871 (646 to 1,165) | 213 (151 to 292) | 1,186 (887 to 1,589) | 139 (97 to 189) |
| **80-84** | 1,005 (721 to 1,375) | 269 (188 to 372) | 1,349 (989 to 1,824) | 161 (112 to 221) |
| **85-89** | 1,093 (793 to 1,451) | 304 (206 to 433) | 1,417 (1,041 to 1,865) | 176 (120 to 249) |
| **90-94** | 1,073 (770 to 1,438) | 292 (197 to 409) | 1,336 (962 to 1,772) | 175 (119 to 245) |
| **>95** | 1,025 (694 to 1,418) | 288 (189 to 418) | 1,215 (828 to 1,690) | 184 (118 to 267) |
| **Cataract** | | | | |
| **50-54** | 113 (65 to 180) | 61 (36 to 94) | 124 (69 to 204) | 167 (110 to 238) |
| **55-59** | 178 (108 to 268) | 91 (58 to 136) | 208 (125 to 318) | 229 (161 to 312) |
| **60-64** | 306 (183 to 462) | 153 (102 to 223) | 361 (212 to 551) | 315 (232 to 427) |
| **65-69** | 611 (379 to 894) | 276 (187 to 399) | 702 (426 to 1,050) | 475 (346 to 635) |
| **70-74** | 1,123 (710 to 1,666) | 506 (350 to 696) | 1,263 (782 to 1,924) | 761 (569 to 985) |
| **75-79** | 1,891 (1,282 to 2,737) | 918 (636 to 1,263) | 2,095 (1,422 to 3,104) | 1,245 (912 to 1,631) |
| **80-84** | 2,746 (1,890 to 3,997) | 1,600 (1,155 to 2,134) | 3,036 (2,033 to 4,520) | 1,991 (1,500 to 2,584) |
| **85-89** | 3,610 (2,515 to 4,986) | 2,634 (1,982 to 3,509) | 3,965 (2,743 to 5,563) | 3,029 (2,348 to 3,945) |
| **90-94** | 4,228 (2,856 to 5,921) | 3,739 (2,851 to 4,886) | 4,643 (3,125 to 6,538) | 4,169 (3,242 to 5,348) |
| **>95** | 4,543 (2,938 to 6,651) | 5,405 (3,881 to 7,247) | 4,977 (3,226 to 7,254) | 5,933 (4,448 to 7,701) |
| **Diabetic retinopathy** | | | | |
| **50-54** | 52 (28 to 84) | 174 (125 to 234) | 37 (18 to 64) | 110 (70 to 165) |
| **55-59** | 74 (41 to 115) | 234 (170 to 312) | 59 (32 to 96) | 142 (90 to 211) |
| **60-64** | 97 (56 to 151) | 283 (206 to 376) | 83 (45 to 133) | 159 (105 to 233) |
| **65-69** | 123 (74 to 190) | 322 (224 to 443) | 109 (62 to 172) | 161 (105 to 245) |
| **70-74** | 147 (89 to 227) | 374 (246 to 535) | 133 (77 to 211) | 166 (103 to 252) |
| **75-79** | 176 (107 to 273) | 439 (275 to 653) | 161 (97 to 253) | 179 (106 to 286) |
| **80-84** | 191 (116 to 301) | 491 (309 to 742) | 178 (106 to 287) | 196 (118 to 317) |
| **85-89** | 193 (124 to 284) | 513 (321 to 773) | 186 (118 to 280) | 208 (127 to 324) |
| **90-94** | 182 (116 to 270) | 523 (318 to 779) | 182 (116 to 273) | 222 (130 to 343) |
| **>95** | 168 (104 to 257) | 556 (321 to 867) | 171 (106 to 266) | 248 (139 to 399) |
| **Glaucoma** | | | | |
| **50-54** | 5 (2 to 9) | 7 (3 to 11) | 5 (2 to 9) | 17 (9 to 27) |
| **55-59** | 17 (10 to 27) | 21 (13 to 33) | 19 (11 to 31) | 54 (35 to 78) |
| **60-64** | 39 (22 to 62) | 49 (30 to 73) | 46 (26 to 73) | 110 (73 to 157) |
| **65-69** | 74 (45 to 114) | 96 (63 to 138) | 89 (53 to 139) | 185 (130 to 251) |
| **70-74** | 118 (71 to 184) | 181 (124 to 253) | 145 (86 to 232) | 297 (217 to 397) |
| **75-79** | 177 (111 to 272) | 342 (230 to 470) | 217 (136 to 337) | 499 (354 to 670) |
| **80-84** | 233 (148 to 357) | 592 (413 to 814) | 283 (179 to 436) | 796 (567 to 1,070) |
| **85-89** | 281 (188 to 408) | 933 (650 to 1,260) | 336 (222 to 498) | 1,189 (849 to 1,593) |
| **90-94** | 303 (196 to 465) | 1,244 (887 to 1,673) | 360 (232 to 551) | 1,602 (1,160 to 2,136) |
| **>95** | 309 (190 to 467) | 1,711 (1,190 to 2,378) | 368 (225 to 559) | 2,260 (1,604 to 3,130) |
| **Age-related macular degeneration** | | | | |
| **50-54** | 4 (2 to 7) | 7 (3 to 13) | 4 (2 to 7) | 10 (5 to 18) |
| **55-59** | 11 (6 to 17) | 20 (11 to 33) | 13 (7 to 20) | 25 (14 to 40) |
| **60-64** | 23 (13 to 36) | 42 (24 to 65) | 29 (16 to 45) | 43 (25 to 65) |
| **65-69** | 40 (24 to 61) | 72 (43 to 111) | 51 (30 to 80) | 62 (38 to 92) |
| **70-74** | 59 (36 to 90) | 114 (69 to 177) | 78 (46 to 122) | 84 (51 to 127) |
| **75-79** | 82 (52 to 124) | 178 (108 to 274) | 108 (68 to 164) | 116 (71 to 176) |
| **80-84** | 100 (64 to 155) | 258 (163 to 388) | 131 (83 to 207) | 156 (97 to 239) |
| **85-89** | 115 (77 to 171) | 355 (228 to 529) | 149 (99 to 222) | 208 (134 to 312) |
| **90-94** | 119 (77 to 174) | 425 (284 to 642) | 154 (100 to 224) | 259 (170 to 390) |
| **>95** | 117 (72 to 178) | 534 (348 to 811) | 153 (93 to 229) | 347 (224 to 523) |
| **Other causes of visual loss** | | | | |
| **50-54** | 85 (50 to 132) | 107 (68 to 154) | 90 (52 to 140) | 213 (147 to 288) |
| **55-59** | 126 (77 to 191) | 136 (86 to 201) | 142 (85 to 219) | 260 (184 to 350) |
| **60-64** | 192 (118 to 281) | 200 (134 to 286) | 230 (139 to 342) | 317 (233 to 424) |
| **65-69** | 300 (185 to 442) | 310 (214 to 435) | 377 (225 to 555) | 391 (285 to 520) |
| **70-74** | 375 (235 to 559) | 504 (360 to 681) | 483 (301 to 733) | 517 (382 to 666) |
| **75-79** | 414 (259 to 640) | 811 (548 to 1,125) | 537 (335 to 814) | 706 (499 to 972) |
| **80-84** | 553 (359 to 860) | 1,289 (930 to 1,736) | 699 (455 to 1,093) | 1,025 (748 to 1,392) |
| **85-89** | 776 (484 to 1,211) | 1,900 (1,349 to 2,578) | 947 (598 to 1,469) | 1,453 (1,022 to 1,975) |
| **90-94** | 1,316 (835 to 1,967) | 3,016 (2,236 to 3,961) | 1,547 (978 to 2,289) | 2,227 (1,627 to 2,960) |
| **>95** | 1,956 (1,151 to 3,010) | 4,829 (3,322 to 6,530) | 2,255 (1,322 to 3,466) | 3,535 (2,316 to 4,878) |

95% UI, 95% uncertainty interval

**References**

1. Vos T, Lim SS, Abbafati C, et al. Global burden of 369 diseases and injuries in 204 countries and territories, 1990–2019: a systematic analysis for the Global Burden of Disease Study 2019. *The Lancet* 2020; **396**(10258): 1204-22.
